# Functional characterization of OR51B5 and OR1G1 in human lung epithelial cells as potential drug targets for non-type 2 lung diseases

**DOI:** 10.1007/s10565-024-09935-9

**Published:** 2024-11-13

**Authors:** Noha Awad, Daniel Weidinger, Lea Greune, Juliane Kronsbein, Natalie Heinen, Saskia Westhoven, Stephanie Pfaender, Christian Taube, Sebastian Reuter, Marcus Peters, Hanns Hatt, Anke Fender, Jürgen Knobloch

**Affiliations:** 1 Medical Clinic III for Pneumology, Allergology and Sleep Medicine, Bergmannsheil University Hospital, Ruhr-University Bochum, Bürkle-de-La-Camp-Platz 1, 44789 Bochum, Germany; 2 Department of Forensic Medicine and Clinical Toxicology, Faculty of Medicine, Ain Shams University, Cairo, Egypt; 3 Department of Molecular and Medical Virology, Ruhr-University Bochum, Universitätsstraße 150, 44801 Bochum, Germany; 4 Research Unit: Emerging Viruses, Leibniz Institute of Virology (N63), Martinistraße 52, 20251 Hamburg, Germany; 5 Institute of Virology and Cell Biology, University of Lübeck, Ratzeburger Allee 160, 23562 Lübeck, Germany; 6 Department of Pulmonary Medicine, University Medical Center Essen – Ruhrlandklinik, Tüschener Weg 40, 45239 Essen, Germany; 7 Department of Pneumology, Mainz University Medical Center and Mainz Center for Pulmonary Medicine, Mainz, Germany; 8 Department of Molecular Immunology, Ruhr-University Bochum, Universitätsstraße 150, 44801 Bochum, Germany; 9 Department of Cell Physiology ND4/35, Faculty of Biology and Biotechnology, Ruhr-University Bochum, Universitaetsstraße 150, 44801 Bochum, Germany; 10 Institute of Pharmacology, University Hospital Essen, University Duisburg-Essen, Hufelandstr. 55, 45122 Essen, Germany

**Keywords:** Olfactory receptor, Lung epithelial cell, Odorant sensitivity, Inflammation, Therapeutic targets

## Abstract

**Background:**

Hypersensitivity to odorants like perfumes can induce or promote asthma with non-type 2 inflammation for which therapeutic options are limited. Cell death of primary bronchial epithelial cells (PBECs) and the release of the pro-inflammatory cytokines interleukin-6 (IL-6) and IL-8 are key in the pathogenesis. Extra-nasal olfactory receptors (ORs) can influence cellular processes involved in asthma. This study investigated the utility of ORs in epithelial cells as potential drug targets in this context.

**Methods:**

We used the A549 cell line and primary bronchial epithelial cells using air–liquid interface culture system (ALI-PBECs). OR expression was investigated by RT-PCR, Western blot, and Immunofluorescence. Effects of OR activation by specific ligands on intracellular calcium concentration, cAMP, Phospholipase C (PLC), cell viability, and IL-6 and IL-8 secretion were analyzed by calcium imaging, enzyme immunoassays, Annexin V/ propidium iodide -based fluorescence-activated cell staining or by ELISA, respectively.

**Results:**

By screening A549 cells, the OR51B5 agonists Farnesol and Isononyl Alcohol and the OR1G1 agonist Nonanal increased intracellular Ca2 + . OR51B5 and OR1G1 mRNAs and proteins were detected. Both receptors showed a preferential intracellular localization. OR51B5- but not OR1G1-induced Ca2 + dependent on both cAMP and PLC signaling. Farnesol, Isononyl Alcohol, and Nonanal, all reduced cell viability and induced IL-8 and IL-6 release. The data were verified in ALI-PBECs.

**Conclusion:**

ORs in the lung epithelium might be involved in airway-sensitivity to odorants. Their antagonism could represent a promising strategy in treatment of odorant-induced asthma with non-type 2 inflammation.

**Graphical Abstract:**

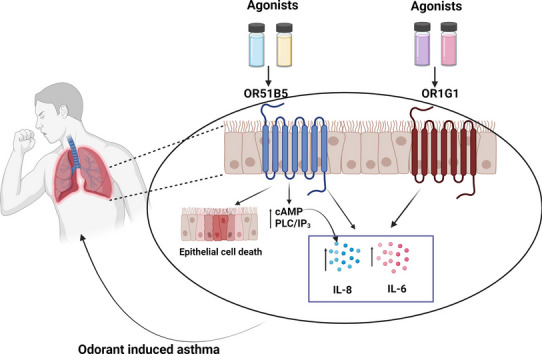

**Supplementary information:**

The online version contains supplementary material available at 10.1007/s10565-024-09935-9.

## Background

Odorants are commonly integrated in many fragranced products such as perfumes, cleaning agents, and personal care items. These substances could exacerbate symptoms in asthmatics. Even healthy subjects report the development of respiratory manifestations by exposure to such products (Flegel and Martin 2015; Steinemann 2018). Odorant-induced respiratory symptoms ranged from mucosal irritation to severe asthma attacks (Steinemann 2018).

Avoidance of exposure to fragranced products stands as the foremost measure to prevent the development of odorant-induced asthma (OIA). Nevertheless, it is not always a feasible strategy. Previous studies reported that a significant proportion of asthmatics are exposed to fragranced products on a weekly basis, either in occupational settings or through passive exposure from scented individuals and surrounding objects (Steinemann 2018).

This indicates the necessity for individuals with fragrance sensitivity to medical intervention, either for acute asthmatic episodes or as long-term therapy to avoid disease progression (Carlsen 2004; Henneberger 2007). Unfortunately, the available treatment options are limited (Gautier and Charpin 2017). This is likely attributed to the fact that more than half of the OIA cases are based on a non-type -2 inflammation (non-T2) (Flegel and Martin 2015).

Recently, the asthmatic population has been categorized into two major groups; non-T2 and T2 phenotypes, based on their clinical presentations and the underlying molecular mechanisms. T2 asthmatic patients show a significant positive response to inhaled corticosteroids. The identification of specific biomarkers for these cases has led to the development of personalized treatments, including monoclonal antibodies targeting immunoglobulin E or type-2 inflammatory molecules such as interleukin IL-4, IL-5, and IL-13 (Hinks et al. 2021). On the contrary, the therapeutic options for non-T2 asthmatics are currently constrained. A significant proportion of these cases exhibit resistance to inhaled steroids. Additionally, biological therapies for non-T2 asthma are limited. This limitation might be attributed to the relative scarcity of studies on possible targets involved in mediating non-T2 asthma (Adrish and Akuthota 2023).

Airway epithelial cells (AECs) are primarily exposed towards inhaled substances that play a pivotal role in OIA (Vandenplas et al. 2014; Xiao et al. 2011). Inhaling irritants strongly contributes to the development of asthma through two main mechanisms: firstly, it induces the death of AECs (Oikonomou et al. 2021), and secondly, it triggers the activation of various inflammatory mediators such as interleukin-17 (IL-17) and interferon-gamma (IFN-γ) (Yang et al. 2021). Elevated IL-17 levels prompt AECs to release IL-6 and IL-8, pro-inflammatory cytokines known to recruit neutrophils, exacerbating the severity of non-T2 asthma (Sze et al. 2020). It was reported that elevated levels of IFN-γ are linked to severe asthmatic attacks that are characterized by reduced responsiveness to steroid treatment. These processes represent central elements in the pathogenesis of non-T2 asthma. Therefore, identification of airway epithelial inflammatory biomarkers could be a promising therapeutic strategy for OIA with non-T2 inflammation (Yang et al. 2021).

Olfactory receptors (ORs) comprise the largest subgroup within the G-protein coupled receptor (GPCR) family, which constitutes targets for roughly 40% of currently available drugs (Santos et al. 2016). Previous literature suggests that the expression of olfactory receptors (ORs) extends beyond the confines of the nasal epithelium; they are found throughout the human body (S. J. Lee et al. 2019; Maßberg and Hatt 2018) and are particularly noteworthy in lung parenchyma, including bronchial epithelial cells (Gu et al. 2014), airway smooth muscle cells (SMCs) (Kalbe, et al. 2016) and alveolar macrophage (Weidinger et al. 2022). Extra-nasal ORs are chemosensory receptors that possess the capability to detect odorants, whether from external sources or internal metabolites. Interestingly, odorants generated endogenously, such as nonanal, 1-propanol, and hexane, were found to be elevated in moderate and severe cases of non-T2 asthma (Savito et al. 2023).

These ORs outside the nasal cavity respond by elevation of intracellular calcium upon odor stimulation (Jovancevic et al. 2017). The modulation of intracellular calcium homeostasis induced by ORs serves as a crucial effector. It orchestrates subsequent cellular events, including the phosphorylation of various kinase enzymes, which, in turn, govern intracellular processes relevant to cellular pathophysiology (Kim et al. 2022; Weber et al. 2017). For example, stimulation of OR1D2 in SMCs by its ligand Bourgeonal results in heightened cellular Ca2 + contraction and triggers the secretion of inflammatory cytokines. (Kalbe et al. 2016). On the contrary, stimulation of OR2AT4 and OR1A2 in alveolar macrophages exhibits an anti-inflammatory effect via diminishing interleukin-8 (IL-8) and IL-6 release (Weidinger et al. 2022). These findings could nominate extra-nasal ORs as a putative therapeutic candidate in cases of OIA.

In this study, we investigated whether airway sensitivity to specific odorants could be regulated through the activation of ORs present in human airway epithelial cells. Consequently, these ORs could potentially serve as novel therapeutic targets for addressing non-T2 asthma triggered by odorous agents.

## Methods

### Cell culture

A549 cells were generously provided by Prof. Dr. Thomas Brüning (IPA, Ruhr-University Bochum). This cell line serves as an in vitro model for human respiratory epithelial cells, specifically for both alveolar and airway epithelium, to evaluate cytotoxicity and (pro)inflammatory responses (Rothen-Rutishauser et al. 2008; Semmler-Behnke et al. 2007; Sengupta et al. 2022). Cells were cultured in Roswell Park Memorial Institute 1640 (RPMI; Sigma-Aldrich, St. Louis, USA, cat: R7638) containing 10% (w/v) fetal bovine serum (FBS, Pan Biotech, Aidenbach, Germany, cat: P30-1506), 100 U/ml Penicillin, 100 µg/ml Streptomycin (Sigma-Aldrich, cat: P4333) 2 mM L-glutamine (Sigma-Aldrich, cat: G7513), 0.25 µg/ml Amphotericin B (Sigma-Aldrich, cat: A2942). A549 were maintained under standard conditions at 37°C with 5% CO2.

The primary bronchial epithelial cells were isolated from postmortem lung transplants (ethics of University Duisburg-Essen 18–8024-BO and 19–8717-BO) using the previously described method by (Schuhenn et al. 2022). Cells were cultured in 0.1% BSA coated flask in keratinocyte-SF-medium, (Gibco, cat: 17005042) supplemented with human epidermal growth factor (Gibco) (2.5 ng/mL), bovine pituitary extract (Gibco) (25 μg/mL), isoproterenol (Sigma-Aldrich, cat: I6504) (1 μM), and 2.5 μg/mL of penicillin/streptomycin, and amphotericin B. Cells were cultured for up to 14 days till around 80% confluency was achieved. 40–60 × 10^3 ^cells were transferred onto trans-well inserts in 12 well plates.

For air–liquid interface (ALI) culture, we used S/D Medium: Bronchial Epithelial Cell Medium basal/ DMEM (ScienCell/Stemcell) medium, supplied with the following components: 1% (P/S) Penicillin/ Streptomycin (Sigma-Aldrich, cat: P4333), 1 M HEPES (Gibco, cat: 15,630,056), 1% Bronchial Epithelial Cell Growth Supplement (Provitro, cat: SC-3262), (5 mM) EC23 (Tocris, cat no: 4011). Cells were grown under submerged medium (liquid–liquid interface, LLI) until they reached 100% confluence. To trigger differentiation of the cells, the apical surface of culture was exposed to ambient air (air–liquid interface, ALI) for 7–14 days. We followed up transepithelial resistance (TEER) and cilia formation as an indicator of epithelial barrier formation and the fully differentiated ALI-primary bronchial epithelial cells (ALI-PBECs) (Leung et al. 2020). ALI culture method was used with PBECs to mimic normal physiological condition (Leung et al. 2020).

#### RNA isolation and reverse transcriptase PCR

The mRNA from A549 and ALI-PBECs was isolated using the RNeasy Plus Mini Kit (Qiagen, Hilden, Germany, cat: 74134). The DNA-free™ Kit (Thermo Fisher Scientific, Waltham, USA, cat: AM1906) was employed according to the manufacturer's instructions to remove any genomic DNA contamination. mRNA concentration was quantified using a Nanophotometer (NP80, Implen, Munich, Germany), and approximately 100 ng of RNA was transcribed into complementary DNA (cDNA) using the iScript™ cDNA Synthesis Kit (Bio-Rad, Hercules, USA, cat: 1708890) as per the manufacturer's guidelines. A reverse transcriptase negative probe (-RT) was performed to confirm the absence of genomic DNA contamination. Polymerase chain reaction (PCR) was carried out with the HotStarTaq DNA Polymerase Kit (Qiagen, cat: 203203), and specific primers were designed using Primer 3 software (Kõressaar et al. 2018)(Version 4.1). The following primers were used for the PCR (Thermo Fisher Scientific):

- **OR51B5:**
  Forward: 5’ CAATGGCACCCTCCTTCTTC-3’
  reverse: 5’ CAAGCAGAATGCCAGACTCG -3’
- **OR1G1:**
  Forward: 5’ TCAATCCCCTCCTGAGTCTG -3’
  reverse: 5’ GACCACGGAGAGATGAGAGC -3’
- **OR10S1**
  Forward: 5’ GCCTGCTGACTCTGGATGGGAA-3’
  reverse: 5’ GGTGAGGGAGGTGTGGATTGCA-3’
- **OR1A1**
  Forward: 5’ ACCATCTCTTGGGCAGCAAA-3’
  reverse: 5’ GGTTGCCACAGAAGGACAGA-3’

All primers followed the same temperature cycle profile: initial activation for 15 min at 95 °C, then 40 cycles of 30 s at 95 °C, 30 s at 55 °C, 60 s at 72 °C, and 10-min elongation step at 72 °C. The PCR products were assessed using a 1.5% agarose gel enriched with 1 × ROTI GelStain Red (Carl Roth, Karlsruhe, Germany, cat: 09841), and the GenRuler 100 bp DNA Ladder (Thermo Fisher Scientific, cat: SM0241) was used as size reference.

### Western blot

Cell lysates of A549 and ALI-PBECs were collected using RIPA buffer (comprising 150 mM NaCl, 50 mM Tris, 1% Nonidet, 0.1% SDS, and 0.5% Natrium Deoxycholat), supplemented with a protease inhibitor as per the manufacturer’s instructions (Merck, Darmstadt, Germany, cat: 11836153001). For better protein separation, the lysates were mixed with twofold Laemmli buffer (BIO-RAD, USA, cat:1610747) and DTT (20 mM). SDS-PAGE and Western blot experiments were performed according to the manufacturer’s recommendation (Gelis et al. 2016). The membranes were stained with Ponceau S (Sigma), blocked for 1 h at room temperature with 5% non-fat dried milk in TBST (150 mm NaCl, 50 mm Tris–Cl, Tween 20, pH 7.4), followed by incubation with the primary antibody OR51B5 (BIOZOL, USA, cat: A13766) with 1:500 dilution and OR1G1(NOVUS, cat: NBP1-68970) diluted as 1 µg/ml in 2.5% of the blocking solution of each antibody overnight at 4 °C.The membrane was incubated with secondary antibodies conjugated to horseradish peroxidase for 1 h at RT, and the signal was detected using ECL Plus (Amersham Biosciences) in conjunction with the Fusion-SL image acquisition system (Alpha Innotech, Germany). The chemiluminescence signals were analysed using ImageJ software (version 1.4.3.67, Broken Symmetry Software).

### Immunofluorescence

A549 cells were seeded, fixed and permeabilized as described by (Weidinger et al. 2022).To avoid non-specific antibody binding, the cells were blocked for 1 h with a 5% goat serum solution in PBST at RT. Primary antibodies against OR51B5 (BIOZOL, USA, cat: A13766), OR1G1 (NOVUS, cat: NBP1-68970) and the membrane marker E-cadherin (Thermo Fisher Scientific, cat: 13–1700) were applied at dilutions of 1:100, 1:200 and 1:2000, respectively in PBST containing 2.5% goat serum and incubated o/n at 4 °C. On the following day, cells were incubated with fluorophore-coupled secondary antibodies. Goat anti-rabbit (1:1000, Thermo Fisher Scientific, cat: A-11010) and goat anti-mouse antibodies (1:1000, Thermo Fisher Scientific, cat: A-A-11029) were used, along with DAPI 1:10,000 (Thermo Fisher Scientific, cat: D1306), for a 45-min incubation in PBST with 2.5% goat serum at RT. Cells were mounted on a slide using ProLong™ Gold Antifade Mountant (Thermo Fisher Scientific, cat: P36934). Images were captured by Fluorescence Olympus IX83 Microscope with 20 × magnification.

### Odorants

The odorants, Farnesol & Nonanal (Sigma-Aldrich, Bayern, Germany), ​ɣ-Decalactone & 2-ethyl-1-hexanol (Symrise, Holzminden, Germany) and Isononyl alcohol (Henkel, Düsseldorf, Germany) were kindly supplied by Prof. Dr. Hanns Hatt from the Department of Cell Physiology at Ruhr-University Bochum. Odorants were pre-diluted at a 1:10 ratio in DMSO. All experiments were conducted while ensuring DMDO final concentration ≤ 0.2%.

### Single-cell calcium imaging

A total of 1 × 10^5 ^cells were plated in a 35 mm dish and incubated till 70 to 80% confluency. Cells were loaded for 30 min with 7.5 μM Fura 2-AM (Enzo, Farmingdale, USA, cat. ENZ-52006) in 1 ml Ringer solution (140 mM NaCl, 5 mM KCl, 5 mM CaCl2, 2.5 mM MgCl2, and 1 mM HEPES) as described before(Gelis et al. 2016). Detailed experimental approaches were described in the results section. Ca2 + -radiometric imaging was measured as previously described by (Weidinger et al. 2022). Images were acquired at a rate of 1 Hz using the DFC360 FX system (Leica, Wetzlar, Germany), and the Leica Application Suite Advanced Fluorescence (LAS AF) was used to quantify the dual fluorescence intensities at excitation wavelengths of (f 340 nm/ f 380 nm) of each individual cell (Grynkiewicz et al. 1985).

### Stimulation

50 × 10^3^ A549 cells were cultured in 24-Well plates with 500 µl culture medium containing 10% fetal bovine serum (FBS) for 48 h before serum- deprivation in 2% FBS media overnight. Afterward, the cells were stimulated for 24 h with 500 µl culture medium containing Farnesol, Nonanal (each at 10–500 µM) and Isononyl alcohol (300–1000 µM) with or without lipopolysaccharide (LPS, 1 µg/ml, from Salmonella enteritidis, Sigma Aldrich, cat: L7770) and the solvent DMSO (0.1%) served as the control.

ALI-PAECs were cultivated in S/D medium and treated with the concentrations that significantly affect A549 cells without leading to complete cell death: Farnesol, and Nonanal were each used at 100–300 µM and Isononyl alcohol was used at 500–1000 µM. In all experiments, both, the apical and the basal parts were exposed to the odorants: The apical part was incubated in 200 µL S/D medium with odorants. The basal part was incubated in 1 mL S/D medium with odorants. After 6 h the apical medium had to be removed to keep cell integrity. Basal medium was collected after 24 h stimulation.

### ELISA

Supernatants of cell cultures were collected, and the IL-8 and IL-6 concentrations were measured via ELISA (Thermo Fisher Scientific, R&D systems, Minneapolis, USA, cat: 88–8086 and cat: 88–7066 respectively) according to the manufacturer’s protocol.

### Flow cytometry: annexin V/PI assay

A549 and ALI-PBECs were treated as previously mentioned. Cells were stained by incubation in 50 µl Hank's Balanced Salt Solution (HBSS) and 5 µl FITC Annexin V (BioLegend, San Diegeo, USA, cat: 640905) for 15 min away from light at 4°C. Cells were washed one time with 1ml of HBSS to stop the reaction. Two µl of propidium iodide (BioLegend, cat: 421301) were added in 1ml of HBSS just prior to measurement. About of 5,000 cells were analyzed in total within the gated regions using the CyFlow SL (Sysmex Partec, Münster, Germany).

### cAMP assay

To measure intracellular cAMP levels, A549 cells were cultured in a 96-well-plate at a density of 1 × 10^4^ per well for 48h. Afterward, cells were stimulated for 20 min with different concentrations of Farnesol and Nonanal (10–500 µM). The intracellular cAMP concentration was determined using cAMP-GloTM Assay (Promega, Wisconsin, USA, cat: V1501) according to the manufacturer’s guidelines.

### Phospholipase C (PLC) assay

3 × 10^6^ A549 cells were cultured in 12-well plates as mentioned before. Two days later, the cells were stimulated for 20 min with different concentrations of Farnesol and Nonanal (10–500 µM) in PBS containing 20mM HEPES. Cells were harvested by scraping in 50 µl of lysis buffer and lysate by 2–3 cycles of freezing (liquid nitrogen)-thawing procedure. The intracellular PLC enzyme concentration was determined in the supernatant using Colorimetric Phospholipase C Activity Kit (Sigma-Aldrich, USA, cat: CS0011) according to the manufacturer’s protocol.

### Statistical analysis

The results were normalized to the corresponding controls and are shown as mean ± SEM. Statistical significance was tested by Paired t-test for experiments where only two groups were compared and one-way analyses of variance (ANOVA) or Friedman test with post hoc "Two-stage-up method of Benjamini, Krieger and Yekutieli" test when more than 2 groups were compared. Statistical analysis was conducted on GraphPad Prism 8 (GraphPad Software, San Diego, CA, USA). ﻿The cut-off value for statistical significance was set at *P* < 0.05.

## Results

### Farnesol, isononyl alcohol and nonanal triggered a transient increase of intracellular calcium in A549 cells

To identify extra-nasally expressed ORs in lung epithelial cells, we conducted a screening of OR functional expression in A549 cells. Our emphasis was on ORs previously reported in the literature to impact various cellular processes implicated in asthma pathology, including smooth muscle cells (SMCs) contractility, cell apoptosis, proliferation, and inflammatory mediator production (Kalbe et al. 2016; Kim et al. 2022), using ligands commonly present in fragranced products (see supplementary Table 1).

**Fig. 1:**
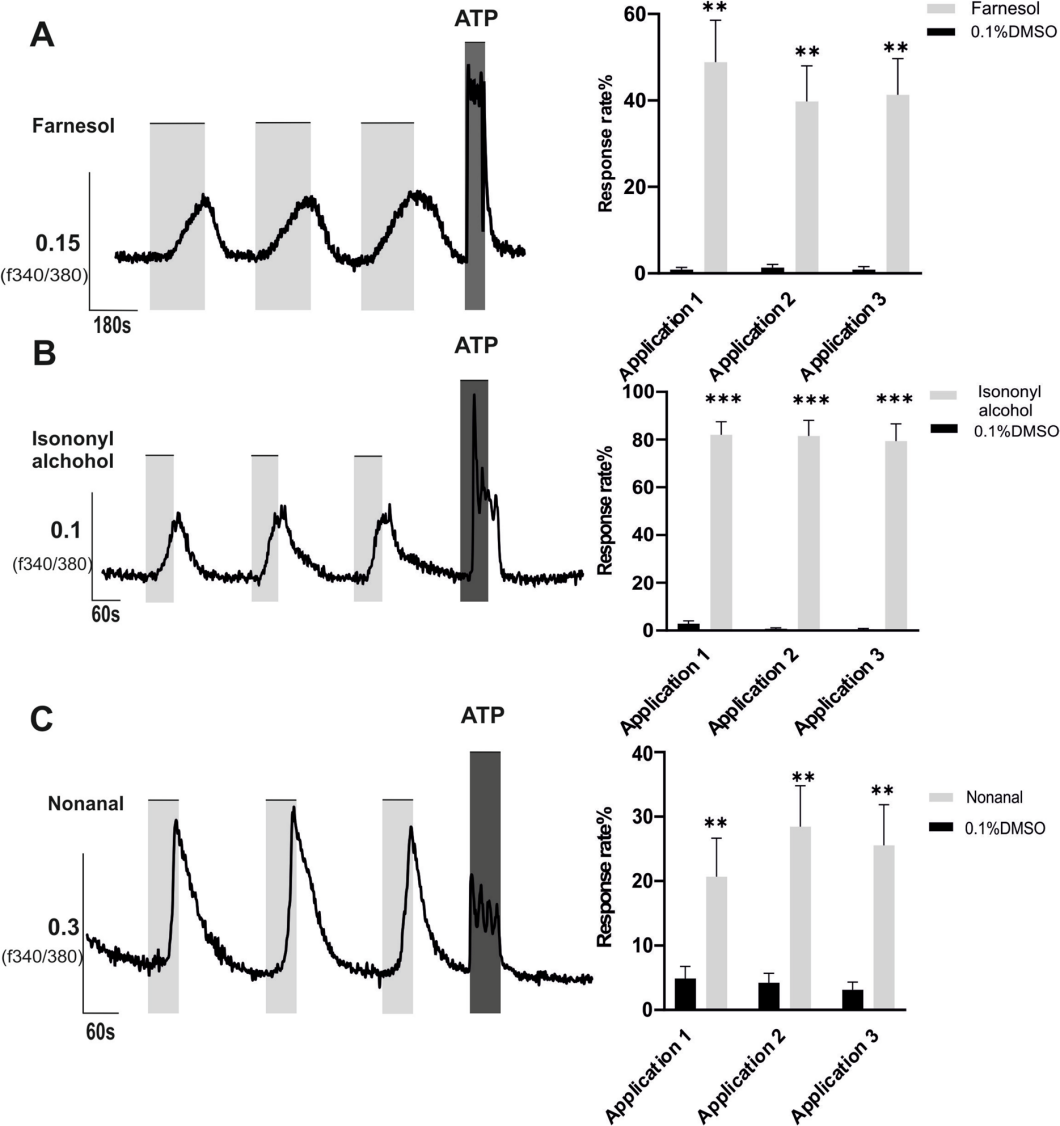
Farnesol, Isononyl alcohol and Nonanal stimulated calcium mobilization in A549 cells. A549 cells were loaded for 30 min with Fura 2-AM followed by measurement of response rate of Ca2 + influx (***f*** 340nm/***f*** 380nm); ratio of responsive cells to the total number of cells, by three times repetitive application of (**A**) Farnesol (300 µM), (**B**) Isononyl alcohol (1 mM) and (**C**) Nonanal (300 µM) compared to the vehicle (DMSO, 0.1%) as indicated. ATP (100 µM) was used as a positive control and was applied after the repetitive addition of the ligands to ensure cell viability. The bars in the graphs on the left side indicate the stimulus duration. Data are shown as mean ± SEM, Technical replicates (TN) = 9. Statistical comparison utilized paired student's t-test referring to the response rate induced by odorant to the DMSO **p ≤ 0.01 and ***p ≤ 0.001

Given previous reports indicating that the functionally active ORs mostly exert their effects by influencing intracellular calcium homeostasis (Jovancevic et al. 2017), we employed real-time fluorometric calcium imaging to monitor changes in intracellular calcium levels following OR activation. Among the investigated odorants (supplementary Table 1), only Farnesol and Isononyl alcohol both agonists for OR51B5 (Manteniotis et al. 2016; Yasi et al. 2019), as well as Nonanal elevated intracellular Ca2 + levels in the A549 cells (Fig. 1). Nonanal is a ligand of more than one OR namely OR1G1 (Park et al. 2020; Sanz et al. 2005),OR1A1 (Park et al. 2020) and OR10S1 (Yasi et al. 2019).

Sequential calcium imaging dose–response experiments were conducted in A549 cells to establish optimal ligand concentrations. Farnesol and Nonanal exhibited a notable proportional increase in the rate of Ca2 + influx at concentrations up to 500 µM compared to solvent controls. However, at higher concentrations, we observed cell detachment indicating for cell death. Consequently, we did not detect a response, even not in the ATP-positive controls that serve as a control for cell vitality. As a result, the dose–response curve did not reach saturation, and estimating EC50 was not possible (Pozdeyev et al. 2016) (refer to Additional Fig. S1). Therefore, concentrations ranging from 10 to 500 µM were utilized in subsequent experiments. Isononyl Alcohol, an alternative agonist for OR51B5, was employed in the following experiments at concentrations up to 1000 µM, as per previous studies (Manteniotis et al. 2016; Tsai et al. 2017).

### OR51B5 and OR1G1 are expressed in A549

**Fig. 2:**
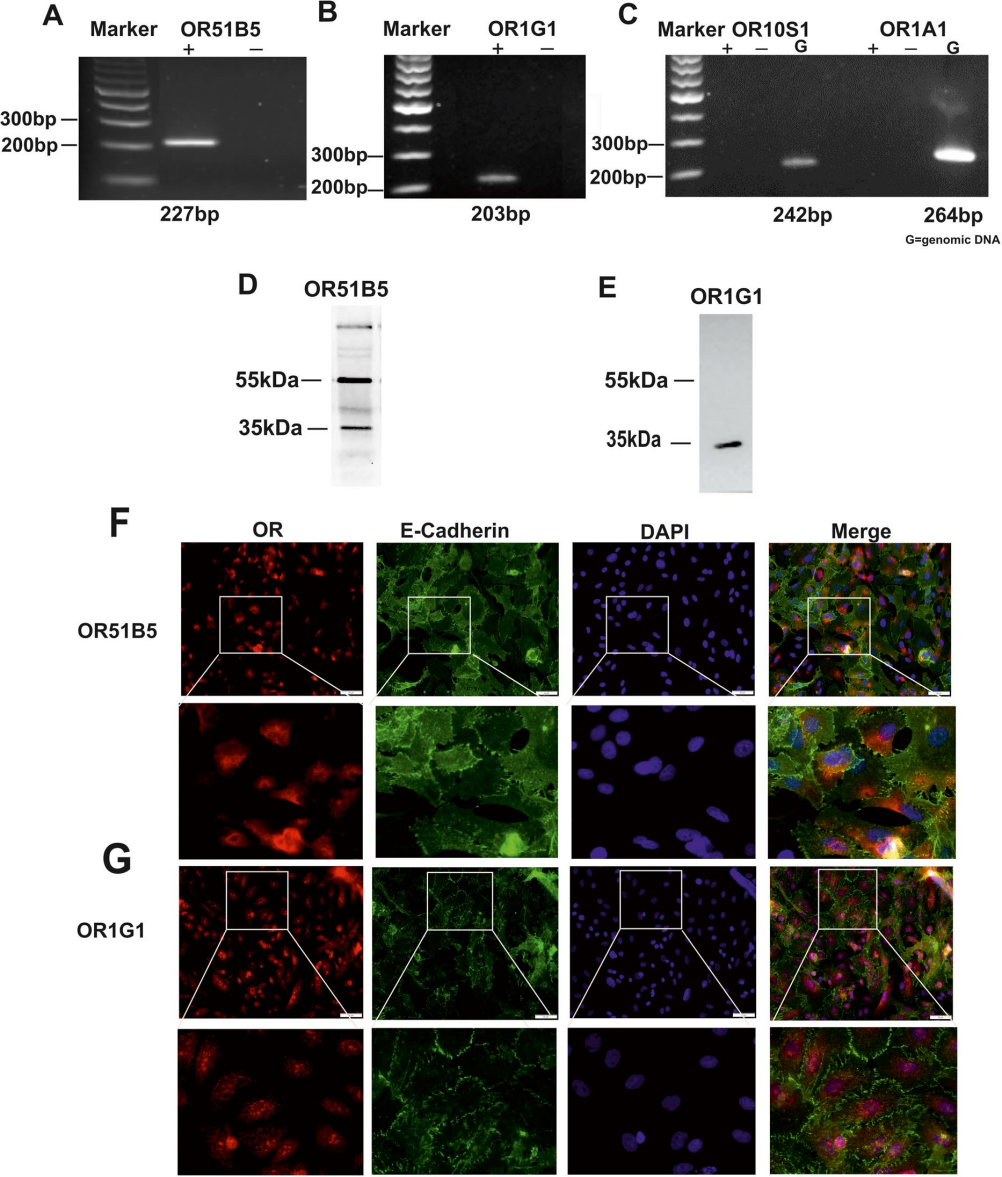
Detection of OR51B5 and OR1G1 expression in A549 cells. RT-PCR demonstrated the presence of (**A**) OR51B5 PCR fragment at 227 bp and (**B**) OR1G1 at 203 bp in A549. (**C**) mRNA transcripts of OR1A1 and OR10S1 were not detected but the genomic DNA was detected for both. The expression of the corresponding proteins was confirmed by (**D**, **E**) Western Blot at the estimated molecular mass of about 35 kDa and (**F**, **G**) immunofluorescence staining, specific antibodies were used against the ORs (red) and the plasma membrane marker E-cadherin (green). The nucleus was stained using 4′,6-Diamidin-2-phenylindol (Dapi; blue). The overlay between the OR (red) and nucleus (blue) staining indicated as violet color in the merged picture. Scale bars: 50 μm and the squares delineate the magnified region. Representative of TN = 3 individual experiments

To investigate OR51B5, OR1G1, OR1A1, and OR10S1 expression in A549, the presence of their mRNAs was assessed by RT-PCR. The mRNAs of OR51B5 and OR1G1 were detected by RT-PCR (Fig. 2 A, B) while mRNA transcripts of OR1A1 and OR10S1 were not detected However, bands representing the genomic DNA served as a positive control and were detected for both (Fig. 2 C). Accordingly, we postulated that OR1G1 is the functionally active olfactory receptor (OR) expressed in A549 cells, potentially mediating the nonanal-induced increase in intracellular Ca^2^⁺ levels. Protein expression of the OR51B5 and OR1G1 was confirmed by Western blot analysis and immunofluorescence staining (Fig. 2 D- G). The latter suggested the intracellular localization of both receptors.

### Farnesol, isononyl alcohol but not nonanal increased the intracellular calcium concentration in A549 via both adenylate cyclase (AC) and phosphoinositide phospholipase C (PLC) dependent pathways

To evaluate the impact of inhibitors on ORs signaling pathways, we first ruled out the potential for (de-) sensitization arising from repeated OR stimulation (Weidinger et al. 2022). This was achieved through application of the agonists three times and by comparing the second and third amplitudes of each experiment to the first amplitude. (refer to Additional Fig. S2).

There are two signaling pathways that are commonly activated by ORs that were previously described. The first is a signaling pathway that is based on the second messenger cAMP which is activated by adenyl cyclase enzyme which led to Ca2 + influx from extracellular space (S. J. Lee et al. 2019; Maßberg and Hatt 2018). The second depends on intracellular Ca2 + release from endoplasmic reticulum (ER) through activation of PLC/ inositol trisphosphate (IP3) pathway (S. J. Lee et al. 2019; Maßberg and Hatt 2018). To investigate the underlying pathway of intracellular Ca2 + increase mediated by Farnesol, Isononyl Alcohol or Nonanal, the following experimental set up were used; we applied the odorants three times each measurement. For the second application, cells were pre-incubated for 3 min with the blocking agent before stimulation. To analyze the effects of the inhibitors, the second application was normalized to the first one (A2/A1).

**Fig. 3:**
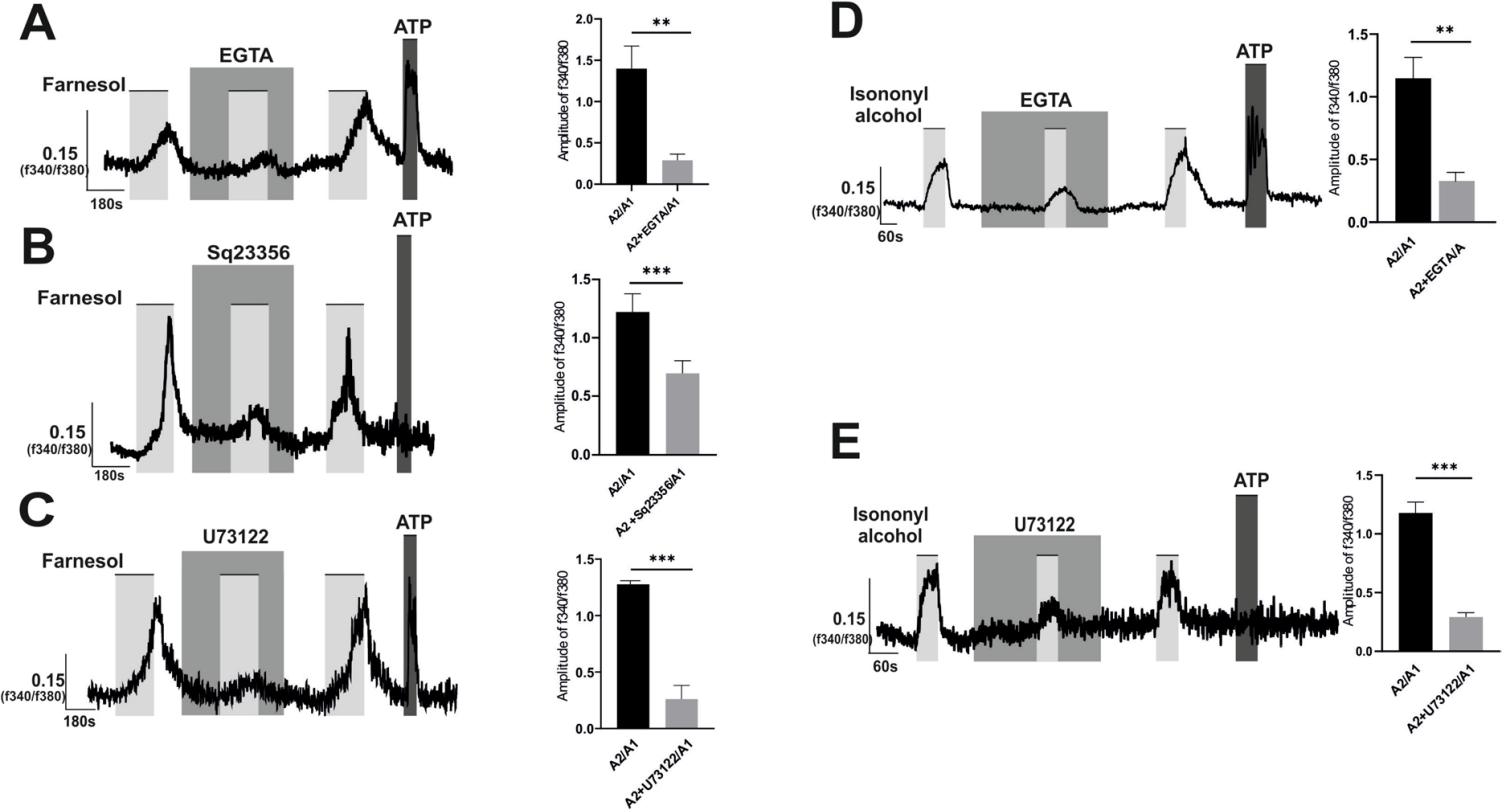
Farnesol-Isononyl alcohol (OR51B5 agonists)-triggered calcium mobilization is mediated by both cAMP/PLC**.** A549 cells were stimulated each measurement three times with (**A**-**C**) Farnesol (300 µM) or (**D**, **E**) Isononyl alcohol (1mM) in presence or absence of the inhibitors; (**A**, **D**) extracellular calcium chelator (EGTA 10mM), (B) Adenylate cyclase inhibitor (SQ 22536 10 µM) or (**C**, **E**) PLC inhibitor (U73122, 2 µM) in the second application, inhibitors were pre-incubated for three minutes before adding odorants in the second application. The bars of all experiments indicate the stimulus duration. Data are shown as mean ± SEM, TN = 7–9. Statistical comparison utilized paired student's t-test referring to the ratio of calcium amplitude between second to first applications in the experimental group (odorants-inhibitors application) vs. the corresponding control group (odorants alone). **p ≤ 0.01; ***p ≤ 0.001

Under Ca2 + free conditions, using calcium-free ringer, the Ca2 + chelator EGTA partially but significantly reduced the Farnesol induced Ca2 + response (Fig. 3A). This indicates that extracellular Ca2 + is a source of OR51B5 induced calcium increase. Furthermore, we analyzed the potential role of AC-cAMP in the OR51B5 cascade. The AC inhibitor SQ 22536 (Manteniotis et al. 2016) partially diminished the Farnesol-induced Ca2 + response (Fig. 3B), indicating the participation of the AC/cAMP pathway in Farnesol-OR51B5 signaling. However, the incomplete reduction in Ca2 + amplitude prompted an investigation into the PLC/IP3 cascade. Treatment with the PLC inhibitor, U73122 (Weidinger et al. 2021), also resulted in a significant yet incomplete reduction in the Ca2 + response (Fig. 3C). Furthermore, when these inhibitors were applied during stimulation with Isononyl Alcohol, the findings supported the conclusion that both the AC and PLC signaling cascades underlie the OR51B5-mediated Ca2 + response (Fig. 3D,E).

Farnesol significantly elevated cAMP and PLC levels (Fig. 5A, C). Forskolin and Carbachol served as positive controls in the cAMP and PLC assays, respectively. Forskolin, a cell-permeable AC activator, augments intracellular cAMP levels (Jin et al. 2023). On the other hand, Carbachol, a cholinergic muscarinic agonist, enhances PLC activity in epithelial cells (Zhang et al. 1995).

**Fig. 4:**
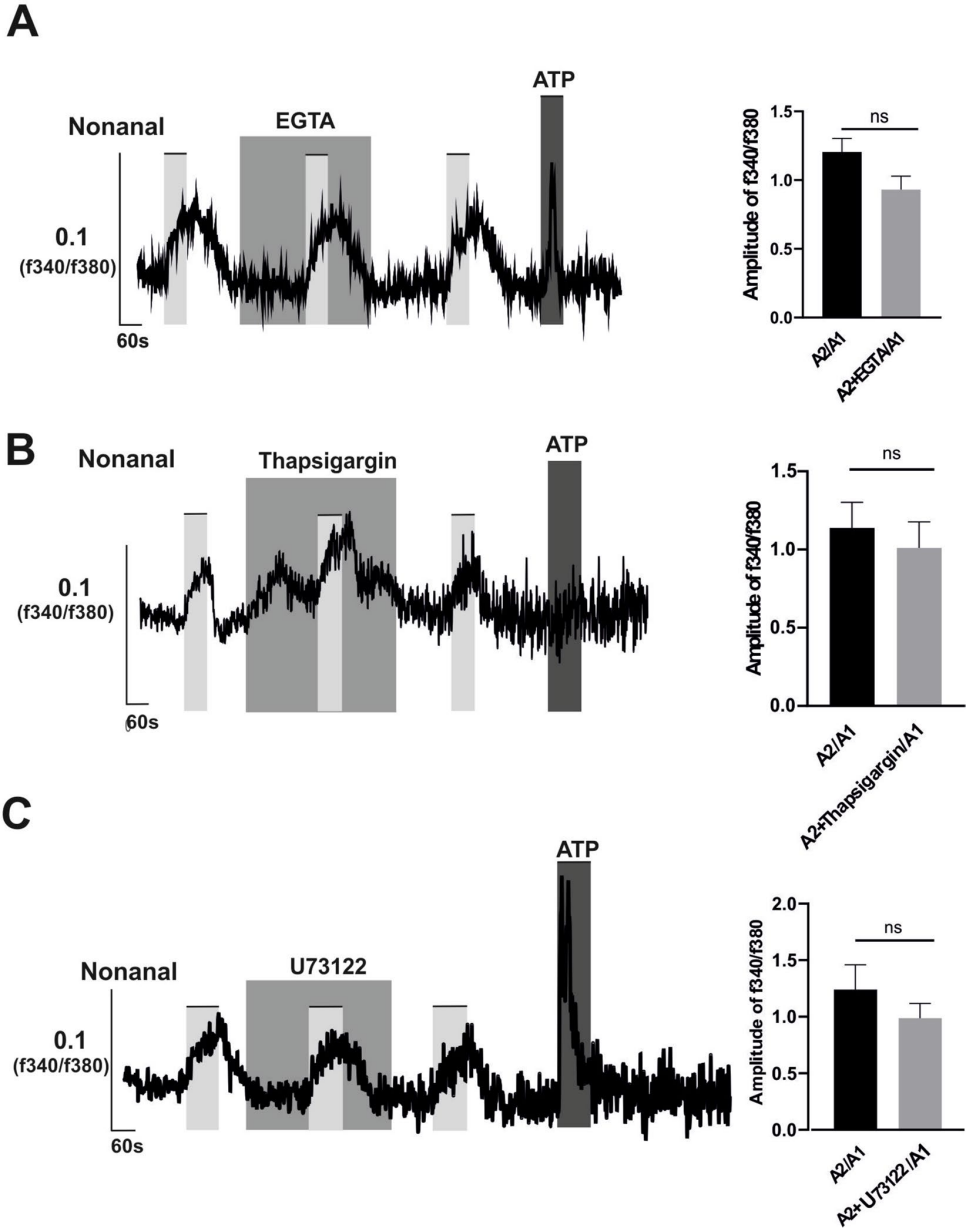
Nonanal, OR1G1 agonist, induced intracellular calcium increase is not from extracellular Ca2 + or intracellular ER**.** A549 cells were stimulated each measurement three times with Nonanal (300 µM) in presence or absence of (**A**) (EGTA 10mM), (**B**) endoplasmic reticulum Ca2 + ATPase inhibitor (Thapsigargin, 5 µM) or (**C**) PLC inhibitor (U73122, 2 µM) in the second application, inhibitors were also pre-incubated for three minutes before adding odorants. The bars on graphs of the left side indicate the stimulus duration. Data are shown as mean ± SEM, TN = 6–9. Statistical comparison utilized paired student's t-test referring to the ratio of calcium amplitude between second to first applications in the experimental group (odorants-inhibitors application) vs the corresponding control group (odorants alone). ns = non-significant

The different signaling effectors of the olfactory cascade were analyzed also with Nonanal. Pre-incubation of EGTA with Nonanal under Ca2 + free conditions did not significantly affect Nonanal-triggered Ca2 + (Fig. 4A), suggesting that the responses induced by Nonanal are independent of extracellular calcium. We further investigated the involvement of internal calcium stores, particularly those within the endoplasmic reticulum (ER). To exclude extracellular calcium integration, all the following experiments were conducted using Ca2 + -free ringer.

**Fig. 5:**
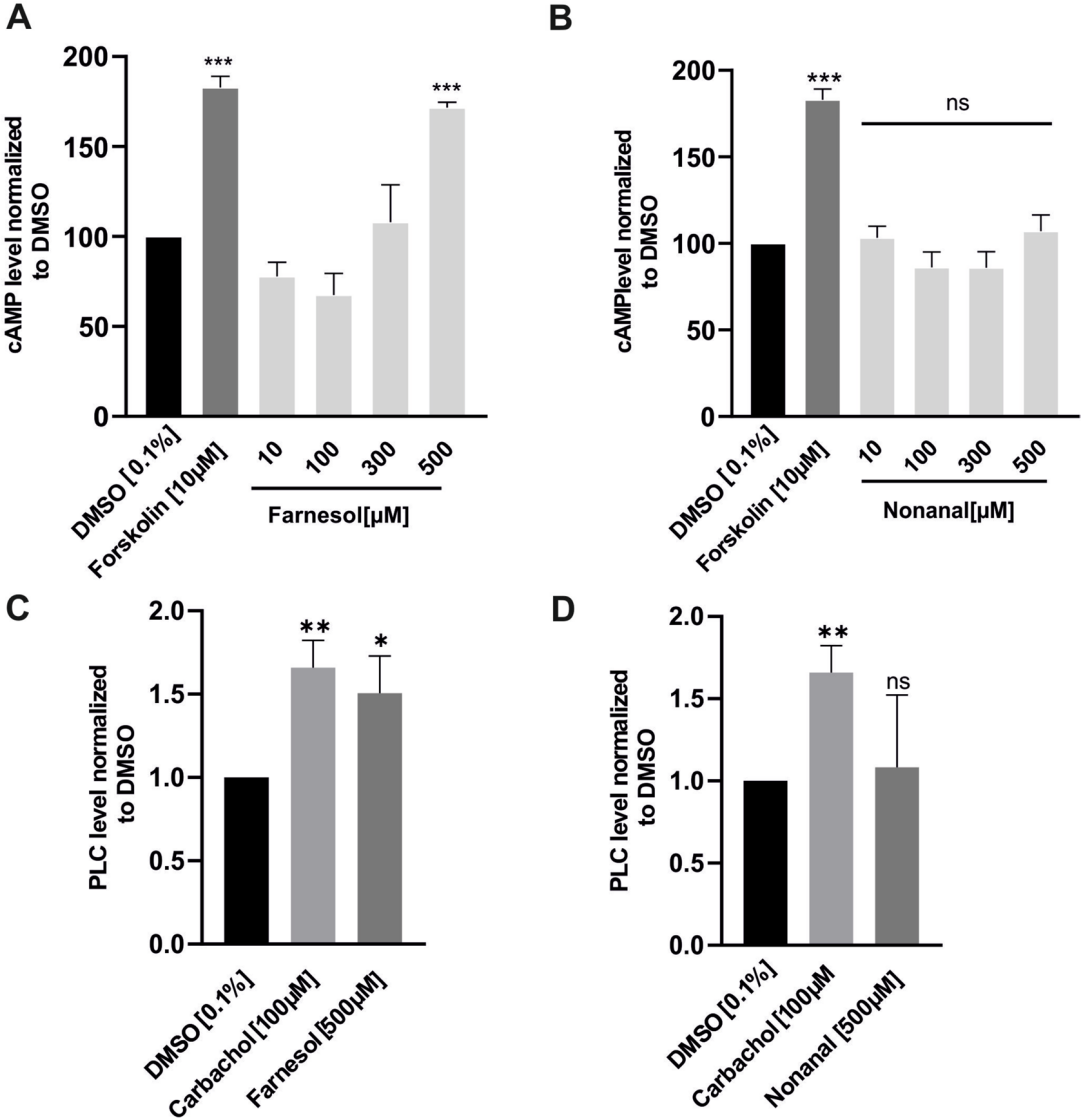
Farnesol but not Nonanal increases the intracellular cAMP and PLC levels. A549 were stimulated with (**A**, **C**) Farnesol, (**B**, **D**) Nonanal or DMSO solvent for 20 min. Concentrations are indicated in the graph. Forskolin and Carbachol are positive controls in cAMP and PLC assay experiments respectively. Data are shown as mean ± SEM, TN = 4–5. Relative changes in (**A**, **B**) cAMP or (**C**, **D**) PLC vs. DMSO 0.1% (negative control) were analyzed by using One-way ANOVA test with post hoc “Two-stage-up method of Benjamini, Krieger, and Yekutieli,”. *p ≤ 0.05 and **p ≤ 0.01 and *** *p* ≤ 0.001. ns = non-significant

Depletion of intracellular ER stores using the ER Ca2 + ATPase inhibitor Thapsigargin (Weber et al. 2017) did not significantly impact the OR1G1-induced Ca2 + increase (Fig. 4B). Additionally, U73122 did not diminish the effect of Nonanal on Ca2 + levels (Fig. 4C), indicating that phospholipase C (PLC) is not involved as a secondary messenger responsible for Ca2 + release from the ER (Weber et al. 2017). Furthermore, Nonanal did not elevate cAMP and PLC levels in the enzyme immunoassay experiments (Fig. 5B, D).

### Stimulation of OR51B5 and OR1G1, both reduces A549 cell viability

The cytotoxic effects of Farnesol, Isononyl alcohol and Nonanal on A549, were assessed using Trypan Blue Dye exclusion assay. The results showed a significant reduction of cell viability in a concentration-dependent manner (Fig. S3).

**Fig. 6:**
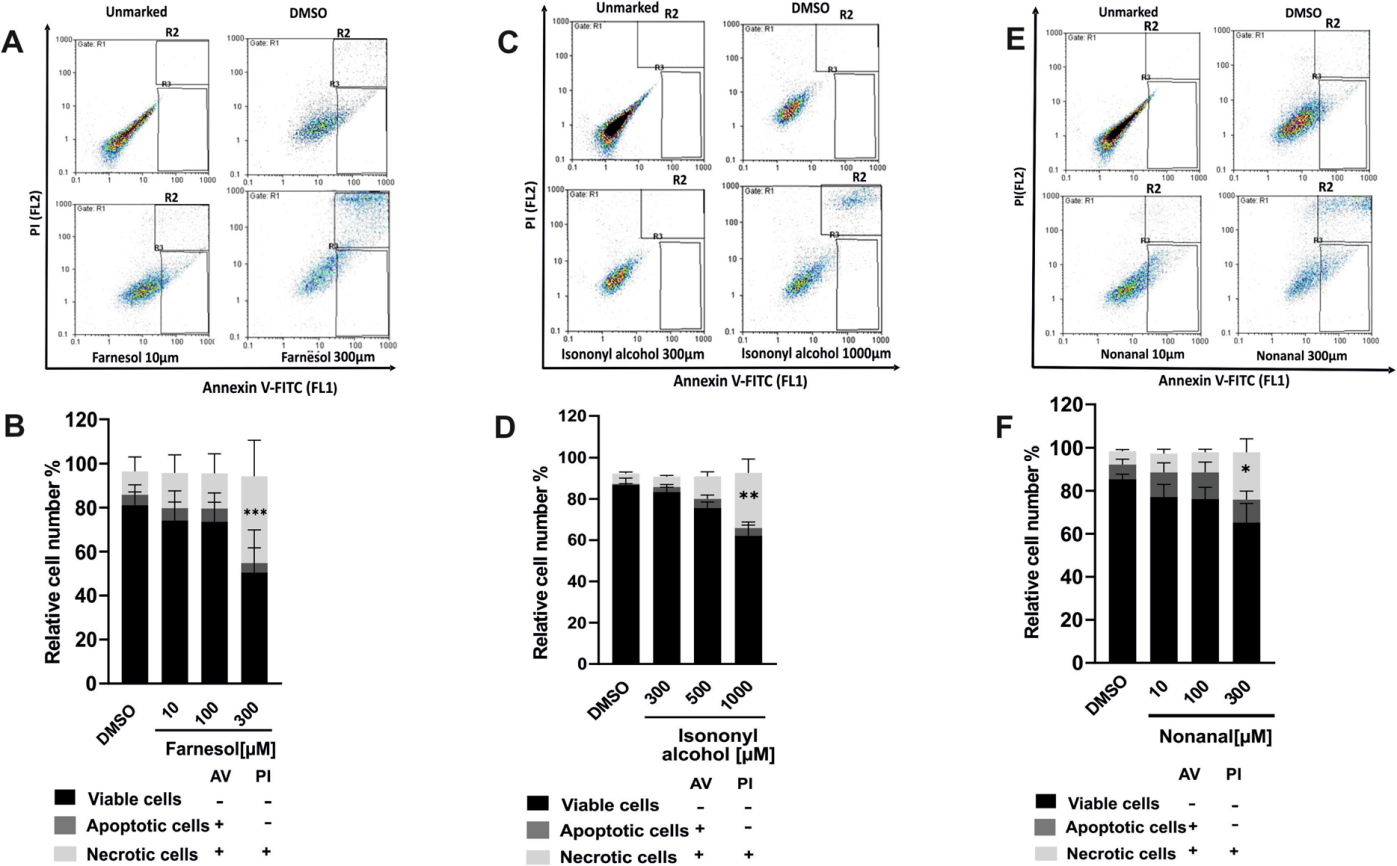
Stimulation of OR51B5 and OR1G1 reduces A549 cell viability. A549 cells were exposed for 24h to increasing concentrations of (**A**, **B**) Farnesol, (**C**, **D**) Isononyl alcohol or (**E**, **F**) Nonanal or solvent DMSO (0.1%). Proportions of viable, apoptotic, and necrotic cells were determined by flow cytometry. Relative cell counts were calculated in comparison to the total cell population. Cells staining positive for Annexin V (AV +) were indicative of early apoptosis and were denoted in the lower right quadrant of the chart (R3), represented by the dark grey portion in the accompanying graphs. AV/propidium iodide (PI) double-positive staining, (AV + , PI +), signified necrotic cells and was in the upper right quadrant of the chart (R2), depicted by the light grey section of the graphs. Viable cells, defined as those unstained by AV and PI (AV − , PI −), were found in the lower left quadrant of the chart and were represented by the black bars in the graphs. Data are shown as mean ± SEM, TN = 5–7. Statistical significance vs. DMSO (0.1%) utilized One-way ANOVA. test with post hoc “Two-stage-up method of Benjamini, Krieger, and Yekutieli,”. *p ≤ 0.05, **p ≤ 0.01 and ***p ≤ 0.001

Flow cytometry analysis was additionally employed to analyze cell viability using Annexin V/Propidium Iodide (AV/PI) staining. Treatment with the OR51B5 agonists Farnesol or Isononyl alcohol or with the OR1G1 agonist Nonanal, resulted in a significant increase in AV/PI positive cells number indicating necrosis (Fig. 6).

### Activation of OR51B5 and OR1G1 in A549 cells augment the secretion of the pro-inflammatory cytokines IL-8 and IL-6

**Fig. 7:**
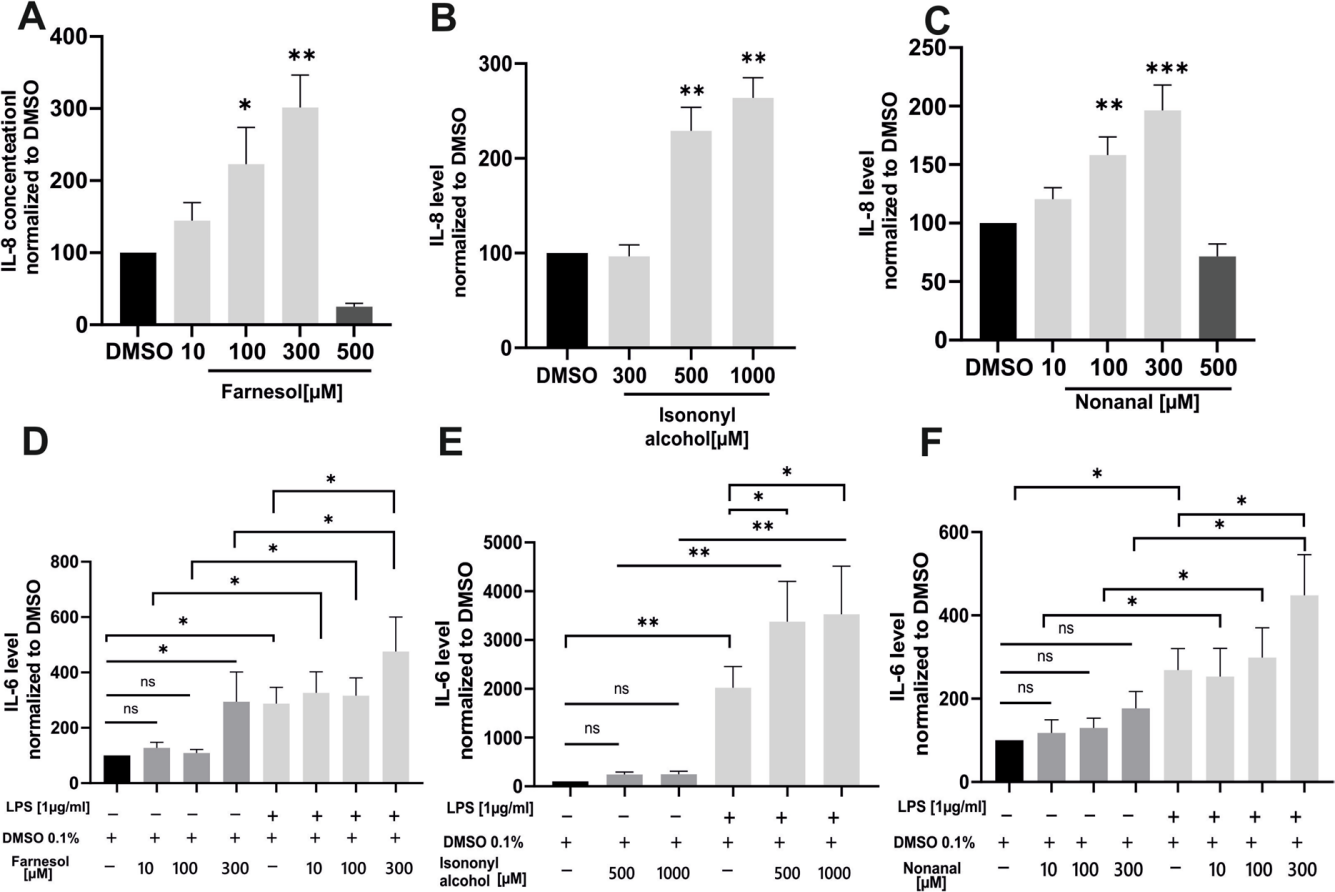
Farnesol, Isononyl alcohol and Nonanal induced IL-8 and IL-6 secretion. A549 cells were treated for 24 h with increasing doses of the odorants; (**A**) Farnesol, (**B**) Isononyl alcohol, (**C**) Nonanal or DMSO solvent. IL-6 experiments cells were stimulated with (**D**-**F**) the same odorants, solvent ± LPS (1 µg/ml). Concentrations are indicated in the graphs. IL-8 and IL-6 in cell culture supernatants was quantified by commercial ELISA. Data were normalized to solvent controls (DMSO) and shown as mean ± SEM. Data significance of agonists vs. (**A**-**C**) DMSO alone or (**D**-**F**) the odorant/vehicle ± LPS was analyzed using One-way ANOVA test with post hoc “Two-stage-up method of Benjamini, Krieger, and Yekutieli,”. *p ≤ 0.05, **p ≤ 0.01 and ***p ≤ 0.001. TN = 5–15

We next investigated the cellular responses mediated through candidate OR stimulation with their respective ligands. We focused on two commonly implicated pro-inflammatory cytokines, IL-8 and IL-6, which are directly associated with the pathophysiology of non-T2 asthma (Sze et al. 2020). Farnesol, Isononyl Alcohol, and Nonanal induced a dose-dependent increase in IL-8 secretion in A549 cells. However, at higher concentrations of 500µM, Farnesol and Nonanal caused up to 100% cell death, which impaired cytokine release. (Fig. 7A-C). For IL-6, a significant increase was observed only in response to Farnesol, while all three compounds, Farnesol, Isononyl Alcohol, and Nonanal, enhanced IL-6 production in LPS exposed cells (Fig. 7D-F).

### Verification of OR1G1 receptor effects by alternative ligands

**Fig. 8:**
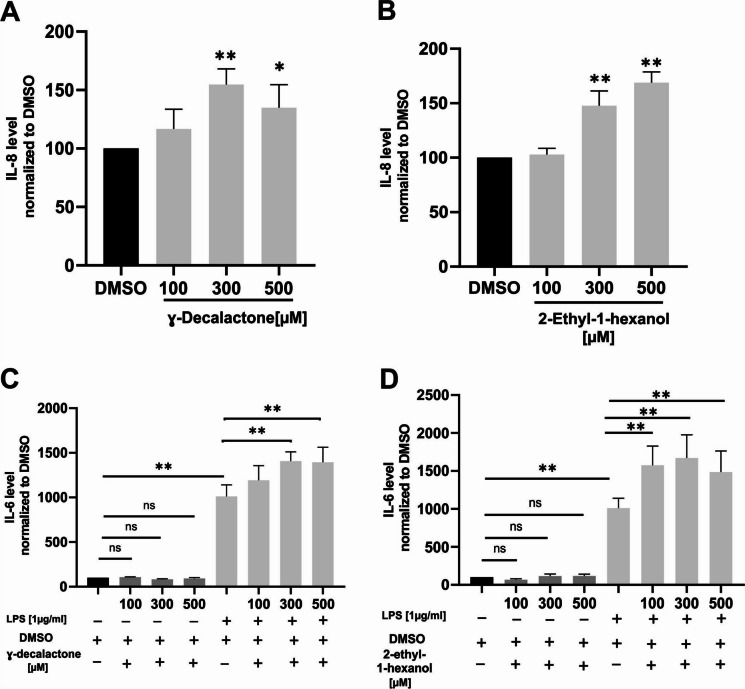
ɣ-Decalactone and 2-ethyl-1-hexanol induced IL-8 and IL-6 secretion. A549 cells were stimulated with ɣ-Decalactone (**A**, **C**) or 2-ethyl-1-hexanol (**B**, **D**), in the presence or absence of LPS, for 24 h with concentrations as indicated in the graphs. IL-8 and IL-6 were measured in culture supernatants by enzyme-linked immunosorbent assay (ELISA). Data have been normalized to solvent controls (DMSO) and are presented as mean ± SEM. TN = 6. Data significance was analyzed using One-way ANOVA test with post hoc “Two-stage-up method of Benjamini, Krieger, and Yekutieli,”. *p ≤ 0.05 and **p ≤ 0.01. ns = non-significant

Because Nonanal is a ligand of more than one OR (Park et al. 2020; Sanz et al. 2005; Yasi et al. 2019). we used OR1G1 alterative ligands (Sanz et al. 2005), ɣ-Decalactone and 2-ethyl-1-hexanol, to verify the inflammatory effects of OR1G1 in A549 cells. Consistent with the effects of Nonanal, both ɣ-Decalactone and 2-ethyl-1-hexanol increased IL-8 and enhanced IL-6 production in LPS-exposed cells (Fig. 8). These findings support the conclusion that the effects of nonanal on cytokine production observed in this study are mediated by OR1G1.

### IL-8 reduction in response to OR51B5 stimulation depends on adenyl cyclase/cAMP pathway

**Fig. 9:**
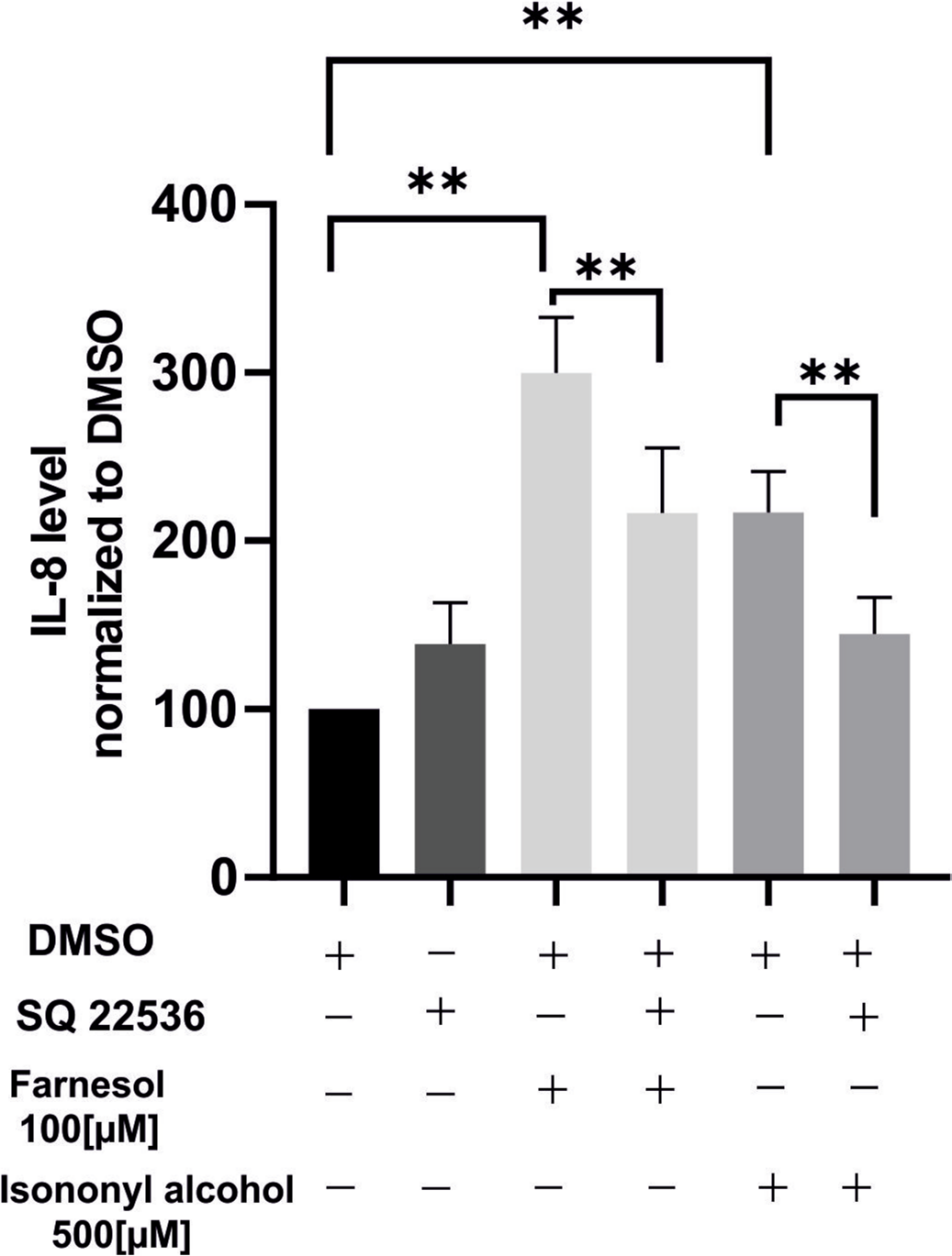
IL-8 induction by Farnesol and Isononyl alcohol is mediated by cAMP. SQ22536 was applied 30 min before stimulation of cells with Farnesol (100µM), Isononyl alcohol (500µM) or DMSO solvent for 24 h.IL-8 concentrations were determined in culture supernatants by ELISA. All data are normalized to solvent controls (DMSO) and presented as mean ± SEM. TN = 6. Data significance of odorants vs. DMSO or the odorant/SQ22536 was analyzed using One-way ANOVA test with post hoc “Two-stage-up method of Benjamini, Krieger, and Yekutieli,”. **p ≤ 0.01

In the previous results, we demonstrated that OR51B5 activation in A549 cells leads to an elevation of cAMP levels, suggesting involvement of cAMP-mediated signaling. Based on this, we hypothesized that the influence of OR51B5 on IL-8 expression is likely associated with this signaling pathway. In support of this, inhibition of adenylate cyclase by SQ22536 effectively counteracted the IL-8 induction caused by Farnesol and Isononyl alcohol (Fig. 9).

### Verification of the data in primary epithelial cells

**Fig. 10:**
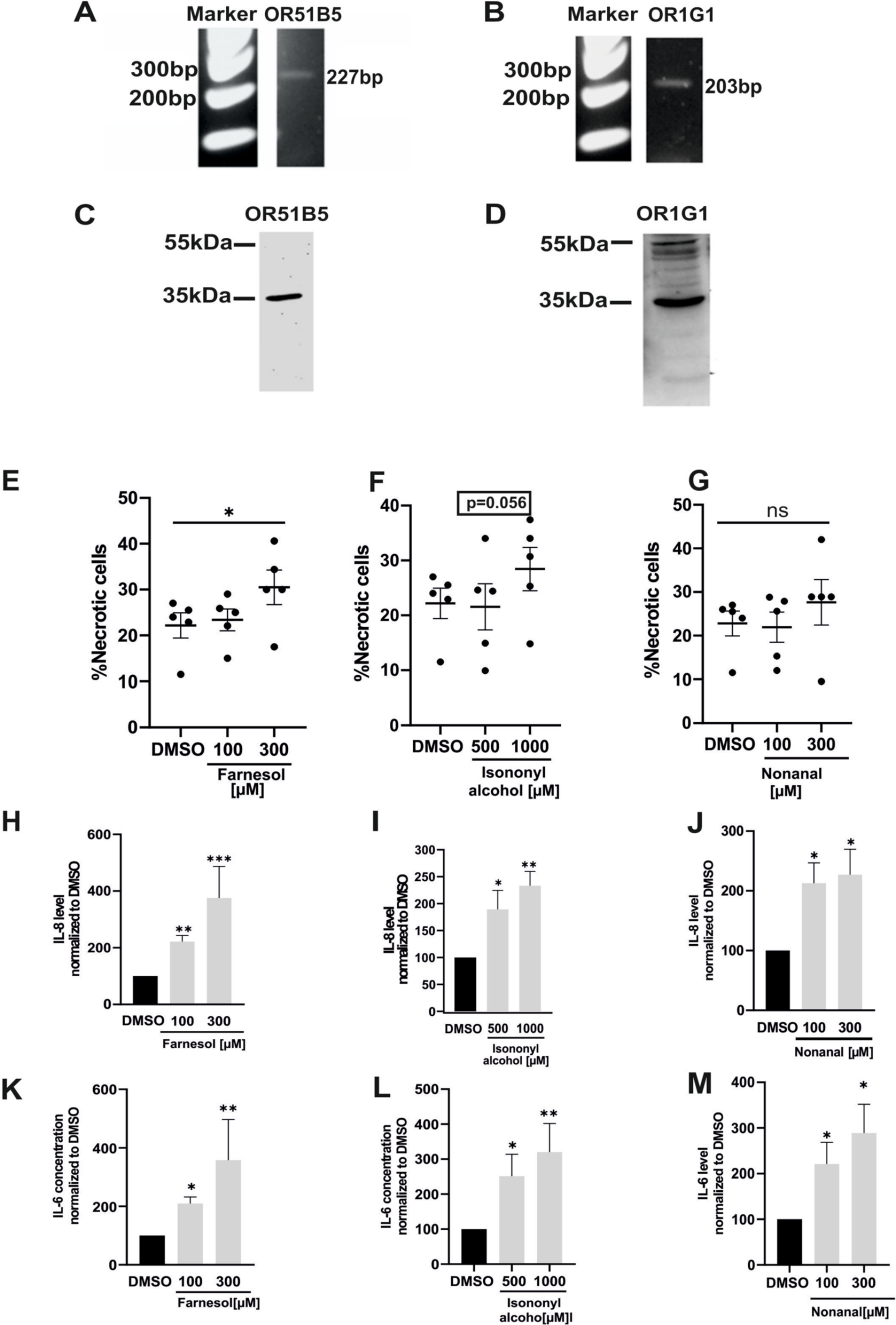
Expression and functional characterization of OR51B5 and OR1G1 ALI-cultured primary epithelial cell**.** RT-PCR revealed the presence of mRNA transcripts of (**A**) OR51B5 and (**B**) OR1G1. Validation of (**C**) OR51B5 and (**D**) OR1G1 protein expression by Western Blot. Evident bands for both receptors at the expected size of 35 kDa are shown. Representative of 3 donors. ALI-PBECs were stimulated with increasing concentrations of (**E**) Farnesol, (**F**) Isononyl alcohol or (**G**) Nonanal or solvent DMSO (0.1%). Proportions of viable, apoptotic, and necrotic cells were determined by flow cytometry using AV/ PI staining. Percentage of (**E**–**G**) necrotic cells were calculated versus total cell count of 5 individual experiments from five donors. ALI-PBECs were stimulated for 6 h with (**H**, **K**) Farnesol, (**I**, **L**) Isononyl alcohol and (**J**, **M**) Nonanal. After 6 h media was removed from apical to preserve cell integrity**.** Supernatant was collected from the basal part after 24 h. Concentrations are indicated in the graphs. IL-8 and IL-6 were measured by ELISA. Experiments were conducted with TN = 6–10 independent samples from six to tent different donors. Data were normalized to solvent controls (DMSO). (**E**-**M**) data are shown as mean ± SEM. Statistical significance vs. DMSO (0.1%) utilized Friedman test with post hoc “Two-stage-up method of Benjamini, Krieger, and Yekutieli,”. *p ≤ 0.05, **p ≤ 0.01 and ***p ≤ 0.001, ns = non-significant

To confirm these findings in a more physiological system, we included ALI-PBECs in our analyses. OR51B5 and OR1G1 expression could be confirmed via RT-PCR (Fig. 10A, B) and Western blot analysis (Fig. 10C, D), respectively.

Flow cytometry analysis using AV/PI staining revealed a notable decrease in cell viability and an increased proportion of necrotic cells following treatment with 300 µM of Farnesol (Fig. 10E), as indicated by the R2 region in the plot graph (Fig. S4). Isononyl Alcohol exhibited a trend toward inducing cell death, although it did not reach statistical significance (Fig. 10F). The OR1G1 ligand Nonanal did not impact the viability of ALI-PBECs under the conditions used (Fig. 10G). All three agonists induced substantial secretion of IL-8 and IL-6 from ALI-PBECs (Fig. 10H-M), suggesting a potential pro-inflammatory role in inflammatory lung diseases with non-T2 phenotype.

## Discussion

This study sheds light on the molecular mechanisms underlying odor sensitivity in individuals with asthma, highlighting the activation of chemosensory olfactory receptors (ORs) expressed in lung epithelial cells. Preliminarily, we conducted our experiments in A549 cell line which has been widely utilized as an in vitro model for human respiratory epithelial cells, particularly both alveolar and airway epithelium to evaluate cytotoxicity and (pro)inflammatory responses (Rothen-Rutishauser et al. 2008; Semmler-Behnke et al. 2007; Sengupta et al. 2022). This is because both airway and alveolar lung epithelial cells react similarly in various lung diseases concerning cytokines and chemokines secretion (Hiemstra et al. 2019). Promising results were subsequently validated in primary lung epithelial cells.

Chronic inflammatory lung conditions like asthma often coincide with heightened sensitivity to odorous stimuli such as perfumes and industrial solvents, which can trigger exacerbations (Gu et al. 2014; Henneberger 2007; Kalbe et al. 2016). Previous research suggests that odorant-induced sensitivity primarily stems from direct irritation of the airway epithelium by inhaled substances (Gu et al. 2014; Vandenplas et al. 2014; Y. Yang et al. 2021). Over 50% of obstructive inflammatory asthma cases exhibit a non-type 2 inflammation phenotype, posing a clinical challenge due to limited therapeutic options (Hinks et al. 2021). To address this challenge, robust biomarkers, and drug targets central to non-type 2 asthma mechanisms must be identified. In this study, we identified OR51B5 and OR1G1 expression in lung epithelial cells and observed an increase in intracellular calcium concentration upon stimulation with their respective agonists, Farnesol, Isononyl Alcohol, or Nonanal (Manteniotis et al. 2016; Park et al. 2020; Sanz et al. 2005; Yasi et al. 2019).

Our investigation into the therapeutic potential of extra-nasal ORs in mitigating odorant-induced airway sensitivity is supported by several factors. Firstly, ORs are specialized receptors to detect volatile odorous stimuli (Gu et al. 2014). Secondly, ORs represent the largest subgroup of GPCR family. GPCRs used as a molecular target for more than one third of all approved drugs (Santos et al. 2016), thus increases the significance of ORs in terms of their pharmaceutical applicability. Recent animal studies and clinical trials also supported the therapeutic utility of extra-nasal ORs (Jimenez et al. 2021; Kalra et al. 2021; Orecchioni et al. 2022; Wu et al. 2021). Thirdly, previous investigations documented the expression of ORs in lung cells and namely in SMCs and neuroendocrine cells in the respiratory epithelium. These receptors play a role in modulating odorant-induced asthma. For example, activation of OR1D2 enhanced airway SMC contraction and triggered the secretion of inflammatory mediators, including IL-8 and granulocyte–macrophage colony-stimulating factor (GM-CSF). Additionally, extra-nasal ORs increase serotonin, and calcitonin gene-related peptide release in pulmonary neuroendocrine-epithelial cells. These findings suggest that hypersensitivity to volatile stimuli in asthma may be attributed to the responsiveness of ORs (Gu et al. 2014; Kalbe et al. 2016). Therefore, targeting extra-nasal ORs in the lung might represent a promising strategy for therapeutic intervention in such cases.

Farnesol, Isononyl Alcohol, and Nonanal, three odorants explored in this study, are common components of various fragranced products like cosmetics, perfumes, detergents, and cleaning agents (Choi et al. 2013; de Araújo Delmondes et al. 2019; Lapczynski et al. 2008; McGinty et al. 2010). Previous data showed that these compounds are inducers of inflammatory reactions and cytotoxic effects (Choi et al. 2013; Gilpin and Maibach 2010; Gu et al. 2014; Schnuch et al. 2004). Furthermore, Nonanal was detected in exhaled breath condensates of non-T2 asthma patients (Savito et al. 2023). Farnesol is also known to be endogenously produced in human liver, kidney, and intestine through the degradation of farnesyl-PP, a metabolite of the cholesterol biosynthetic pathway. Farnesol exerts negative feedback effects on this pathway, leading to apoptosis and cell cycle downregulation (Joo and Jetten 2010; Staines et al. 2004). These odorants exhibit relative specificity towards different olfactory receptors such as OR51B5, OR1G1, OR1A1, and OR10S1(Manteniotis et al. 2016; Park et al. 2020; Yasi et al. 2019). However, in our study, only OR51B5 and OR1G1 expression was detected at both the mRNA and protein levels in both A549 and ALI-PBEC. OR51B5 and OR1G1 have been previously found in various cell types; for example, OR51B5 in keratinocytes (Tsai et al. 2017), K562 leukemia cells (Manteniotis et al. 2016), while OR1G1 was detected in Dermal papilla cells (DPCs) (Park et al. 2020) and primary iliac Enterochromaffin cells (Braun et al. 2007). These ORs were found to modulate various physiological processes through increase of intracellular Ca2 + (Braun et al. 2007; Manteniotis et al. 2016). Therefore, we aimed to investigate the effect of OR51B5 and OR1G1stimulaton with Farnesol, Isononyl alcohol, and Nonanal on A549 and ALI- PBECs. The odorant concentrations used in this study were chosen based on dose–response experiments and are commonly employed in various preclinical in vitro studies investigating olfactory receptors (Cong et al. 2022; Jovancevic et al. 2017; Weidinger et al. 2021) and corresponding or higher odorant concentrations have been used in ex or in vivo experiments and clinical trials (Chéret et al., 2018; Colegio et al., 2014; J. H. Lee et al. 2015; Jimenez et al. 2021; Orecchioni et al. 2022; Pluznick, 2013; Sharma et al., 2023).

Two conventional signaling pathways are associated with the activation of extra-nasal olfactory receptors (ORs). The first pathway involves the activation of adenylate cyclase (AC), which synthesizes the second messenger cyclic adenosine monophosphate (cAMP) from adenosine triphosphate (ATP). cAMP then binds to cyclic nucleotide-gated ion channels, leading to the influx of extracellular calcium ions into the cells (S. J. Lee et al. 2019; Maßberg and Hatt 2018). The second signaling cascade commonly relies on the second messenger IP3, activated by PLC. IP3 triggers the release of intracellular calcium from the ER into the cytosol (S. J. Lee et al. 2019; Maßberg and Hatt 2018). Our investigation revealed that OR51B5 not only increased intracellular calcium levels from extracellular calcium sources by triggering the AC/cAMP-dependent signaling cascade but also from intracellular calcium stores by stimulating the PLC/IP3 signaling pathway. This dual effect of ORs on distinct signaling pathways within the same cell has been previously reported in the literature (Gelis et al. 2016; Weidinger et al. 2021). Regarding OR1G1 activation, Ca2 + imaging results ruled out the involvement of the common pathways previously described with ORs, as depletion of extracellular Ca^2^⁺ had no impact on the observed Ca^2^⁺ influx. We concluded that OR1G1 triggers Ca2 + release from intracellular Ca2 + stores. However, our results suggest that the endoplasmic reticulum (ER) is an unlikely source of this intracellular Ca^2^⁺, as neither U73122 nor Thapsigargin affected the nonanal-induced Ca^2^⁺ release. Similar observations were reported by Kalbe et al. (2017) in OR 2J3 induced Ca2 + increase. Further studies are needed to explore OR1G1-dependent signaling cascade.

Excess bronchial epithelial cell death may contribute to the characteristic airway remodeling in severe refractory asthma (Oikonomou et al. 2021; Sauler et al. 2019). Therefore, we investigated the effect of OR stimulation on cell viability. Our study revealed a notable decrease in the epithelial cell viability following incubation with ligands for both OR51B5 and OR1G1. Consistent with our results, prior research has documented that stimulation of OR51B5 with Isononyl Alcohol inhibits cell proliferation in K562 cells via AC-cAMP-mediated intracellular calcium increase (Manteniotis et al. 2016). Moreover, several studies indicated apoptotic effects of Farnesol in A549 (Miquel et al. 1998; Wang et al. 2003). One of the elucidated mechanisms involves the elevation of cytosolic Ca2 + levels by Farnesol, leading to apoptosis (Joo and Jetten 2010). Here, we could demonstrate that the downstream signaling of OR51B5 in A549 cells involves cAMP-mediated elevation of intracellular Ca2 + level, suggesting that the effect of OR51B5 on cell viability is likely linked to this signaling pathway. Nonanal was also documented to induce apoptosis in A549 cells (Choi et al. 2013), However, the focus of this study was on investigating the downstream signaling pathways that lead to apoptosis in Nonanal-treated cells, rather than identifying the direct target receptor of Nonanal as a ligand (Choi et al. 2013). In this context, a contradictive observation suggested that OR51B5 activation plays an essential role in the survival of human dermal fibroblasts (Hs68 cells) (Son et al. 2021). This disagreement could be explained by the distinct physiological responses in different cell types because the binding of a certain odorant to the same OR could lead to activation of different molecular and cellular mechanisms depending on the cellular phenotype (Manteniotis et al. 2016; Weidinger et al. 2021). Notably, we observed that the cytotoxic effect seems to be stronger with Farnesol than Isononyl alcohol. This suggests that Farnesol is likely a more potent agonist for the OR51B5 receptor. A supportive finding was reported by (Manteniotis et al. 2016) who described Isononyl alcohol as a low-affinity OR51B5 agonist exerting less significant physiological effects.

We extended our investigation to study the impact of odorants on cell viability in primary airway epithelial cell cultures. We used for our experimental setup an air–liquid interface (ALI) model which represents a physiologically relevant culture system (Leung et al. 2020) and serves as a valuable tool for elucidating mechanistic insights into the toxicity of chemical compounds (Djidrovski et al. 2023). Consistent with our observations in the cell line, the results showed higher cytotoxic effects of Farnesol compared to Isononyl alcohol in primary cells. Farnesol significantly augmented the occurrence of cell death, whereas Isononyl Alcohol affected cell viability without reaching statistical significance.

In contrast, nonanal exhibited no noticeable change of cell viability in ALI-PBECs. Differences between cell lines and corresponding primary cells in terms of metabolic pathways and associated biological functions have been reported in previous literature (Pan et al. 2009). Discrepancy of results between A549 and ALI on cell viability could be explained by the previous observation indicating the upregulated extra-nasal ORs in cell line and tumor tissue compared to normal tissue (Chung et al. 2022). Altered expression of extra-nasal ORs was also documented in other pathological conditions such as chronic obstructive lung disease (Gu et al. 2014), neurodegenerative and metabolic disorders (Ferrer et al. 2016; Z. Yang et al. 2023). These receptors may contribute to disease pathogenesis by mediating the release of inflammatory mediators or neuropeptides. Additionally, they could modulate the relative sensitivity of drugs, as observed with chlorpromazine, an antipsychotic, in the treatment of neuropsychiatric diseases (Ansoleaga et al. 2015; Gu et al. 2014). In light with the findings from our present study, which highlight the involvement of OR51B5 and OR1G1 in epithelial cell death and inflammation (as discussed below), further exploration of the potential over-expression of our candidate ORs in airway epithelium could offer a compelling scenario. Such investigations may help elucidate the heightened sensitivity of individuals with asthma to the respective odorants.

IL-8 and IL-6, secreted by airway epithelial cells, are key drivers of non-T2 asthma, particularly through neutrophil recruitment, which contributes to steroid resistance (Sze et al. 2020; Wei et al. 2023). We investigated the role of olfactory receptors OR51B5 and OR1G1 in regulating these cytokines. Activation of OR51B5 and OR1G1 increased IL-8 and IL-6 secretion in A549 cells and ALI-PBECs. However, in A549 cells, IL-6 secretion required co-stimulation with LPS, as these cells alone do not produce detectable levels of IL-6. LPS, a potent inducer of cytokine release (Liu et al. 2018), facilitated IL-6 detection in this cell line. Importantly, co-stimulation with odorants and LPS significantly increased IL-6 secretion compared to LPS stimulation alone, indicating that odorants enhance IL-6 release. This finding is consistent with Wang et al. (1999), who reported that IL-6 secretion is significantly higher in primary bronchial epithelial cells compared to A549 cells.

We identified a signaling pathway in lung epithelial cells that leads to activation of adenylyl cyclase/ cAMP signaling cascade via stimulation of OR51B5. The subsequent Inhibition of cAMP hampered the receptor inducing effect on IL-8 secretion. The production of IL-8 in lung tissue is regulated by p38 MAPK, which has been shown to be activated by protein kinase A in response to elevated cAMP levels (Knobloch et al. 2019; Seo et al. 2022). This signaling cascade contributes to the development of corticosteroid insensitivity in non-T2 inflammation due to IL-8 induced neutrophilic recruitment (Knobloch et al. 2019). Given the current lack of effective anti-inflammatory treatments for non-T2 asthma, largely due to the emergence of steroid resistance (Adrish and Akuthota 2023), our results provide the first indication that targeting IL-8 and IL-6 secretion, either by inhibiting cAMP signaling or through direct antagonism of OR51B5 and OR1G1, could form the basis for developing new classes of anti-inflammatory drugs for patients with (OIA), particularly those with the non-T2 phenotype.

It's worth mentioning here, that there was a marked increase of the cytokines upon treatment of cell line and primary cells with different odorants despite their significant influence on cell viability. It was previously documented that the induced release of interleukins could be either through an active mechanism of the viable cells or a passive process after membrane disruption following cell death (Evavold et al. 2018; Martin-Sanchez et al. 2016). However, we found that application of higher concentration of odorants that resulted in up to 100% cell death did not cause an increase in interleukin levels. This supports that the elevated levels of IL-8 and 6 are mainly through active release from viable A549 and ALI-PBECs rather than that they are a direct consequence of cell death.

Odorants often activate more than one OR (Beito et al. 2024). Therefore, we aimed to verify that the candidate ORs mediate the observed endpoints analyzed in this study. Attempts to knock out OR51B5 and OR1G1in A549 cells using the CRISPR/Cas9 technique were unsuccessful, as it has been shown before for other ORs (Weidinger et al. 2024). We speculate that OR51B5 and OR1G1may possess unusually long half-lives, complicating the knockout approach (Weidinger et al. 2024). Consequently, we adopted an alternative strategy utilizing surrogate ligands. Isononyl alcohol, an OR51B5 ligand (Manteniotis et al. 2016), resembled the endpoint effects of Farnesol on A549 and primary lung epithelial cells. Moreover, the OR1G1 ligands ɣ-Decalactone & 2-ethyl-1-hexanol (Sanz et al. 2005), both, induced induction of IL-8 and IL-6 consistent with the effects observed with Nonanal. Thus, we conclude that OR51B5 and OR1G1 are involved in modulating the patho-physiologically relevant endpoints investigated in this study.

Immunoflourescence staining demonstrated that both receptors were predominantly found intracellularly. This localization was also reported for OR 51E2 in human Melanocytes (Gelis et al. 2016). It's not uncommon for GPCRs to be functionally expressed not only in plasma membranes but also across various intracellular compartments such as the nucleus, endosomes, Golgi apparatus, endoplasmic reticulum, and mitochondria. These receptors have the capacity to initiate downstream cascades, including Ca2 + /cAMP and/or protein kinase signaling, either from cytosolic localization or from organelles (Fasciani et al. 2022; Mohammad Nezhady et al. 2020). This observation could potentially explain why repetitive stimulation of the olfactory receptors (ORs) in our study did not result in (de-) sensitization of Ca2 + release, as one might expect from receptors located in the plasma membrane.

Intracellular G protein-coupled receptors (GPCRs), including olfactory receptors (ORs) (Santos et al. 2016), might represent a promising therapeutic target. Access to these receptors could occur either through passive diffusion across the plasma membrane, particularly by lipophilic ligands, or via specific protein transporters. However, passive diffusion, even for lipophilic compounds, is generally limited. Most ligands that have the potential to diffuse across the membrane primarily rely on transporters for effective delivery to intracellular receptors. These transporters, in conjunction with other carrier proteins, facilitate the precise localization of signaling molecules to specific intracellular organelles. (Fasciani et al. 2022; Mohammad Nezhady et al. 2020). The expression of a drug target intracellularly rather than on the outer plasma membranes may increase the probability of off-target interactions and possible side effect (Babcock and Li 2013). To address this challenge, a strategy has been proposed involving the development of prodrugs that stimulate membrane transporters to release the parent drug at its site of action. This approach aims to circumvent undesired effects and enhance drug efficacy. However, while promising for intracellular therapeutic targets, implementing this strategy poses significant challenges and requires thorough mechanistic and pharmacokinetic studies before it can be considered a viable therapeutic intervention (Mohammad Nezhady et al. 2020; Puris et al. 2022).

In conclusion, this study elucidated the functional expression of OR51B5 and OR1G1 in both A549 cells and primary bronchial epithelial cells. Selective stimulation with their agonists, including Farnesol, Isononyl Alcohol, and Nonanal, resulted in differential effects on intracellular Ca2 + mobilization, cell viability, and secretion of pro-inflammatory cytokines implicated in the pathogenesis of non-type 2 asthma, namely IL-8 and IL-6. These findings suggest that the sensitivity of human airways to odorants commonly found in perfumes and cleaning products may be mediated through the direct activation of these candidate ORs. Thus, the study underscores the potential utility of targeting lung epithelial OR51B5 and OR1G1 as drug targets for odorant-induced asthma with non-type 2 inflammation.

## Supplementary information

Below is the link to the electronic supplementary material.

ESM 1 (DOCX 508 KB)

## Data Availability

All original data related to the study are available from the corresponding author upon request.

## Abbreviations

*AC*: Adenyl cyclase
*AECs*: Airway epithelial cells
*ALI*: Air- liquid interface
*ALI-PBECs*: ALI-primary bronchial epithelial cells
*BSA*: Bovine serum albumin
*Ca*2 +: Calcium
*cAMP*: Cyclic adenosine monophosphate
*ATP*: Adenosine triphosphate
*cDNA*: Complementary DNA
*DAPI*: 4′,6-Diamidin-2-phenylindol
*DMSO*: Dimethyl sulfoxide
*DTT*: Dithiothreitol
*EGTA*: Ethylene glycol-bis (β-aminoethyl ether)-N, N, N′, N′-tetra acetic acid
*ER*: Endoplasmic reticulum
*f*: Fluorescence intensities
*FBS*: Fetal bovine serum
*GPCRs*: G protein-coupled receptors
*GM-CSF*: Granulocyte-macrophage colony-stimulating factor
*HEPES*: N-2-hydroxyethylpiperazine-N'-2-ethanesulfonic acid
*IL*: Interleukin
*IFN-γ*: Interferon gamma
*LLI*: Liquid- liquid interface
*LPS*: Lipopolysaccharide
*non-T2*: Non type 2 asthma
*NSCLC*: Non-small-cell lung cancer
*OIA*: Odorant-induced asthma
*OR*: Olfactory receptor
*PBECs*: Primary bronchial epithelial cells
*PBS*: Phosphate-buffered saline
*PBST*: Phosphate-buffered saline + Triton X-100
*PCR*: Polymerase chain reaction
*PFA*: Paraformaldehyde
*PI*: Propidium iodide
*PLC*: Phospholipase C
*RPMI*: Roswell Park Memorial Institute
*RT-PCR*: Reverse transcriptase PCR
*S/D*: Stem cell medium/ Dulbecco′s Modified Eagle′s Medium
*SDS-PAGE*: Sodium dodecylsulfate polyacrylamide gel electrophoresis
*SEM*: Standard error of mean
*SMCs*: Smooth muscle cells
*T2*: Type 2 asthma
*TEER*: Transepithelial resistance
*TN*: Technical replicates
