# Supplementary material for: Functional characterization of OR51B5 and OR1G1 in human lung epithelial cells as potential drug targets for non-type 2 lung diseases
Source: Cell Biol Toxicol. 2024 Nov 13;40(1):96. doi: 10.1007/s10565-024-09935-9 (PMC11561009; doi:10.1007/s10565-024-09935-9)
Supplement: Supplementary file 1 — (DOCX 508 KB) [file 10565_2024_9935_MOESM1_ESM.docx]

**Supplement**


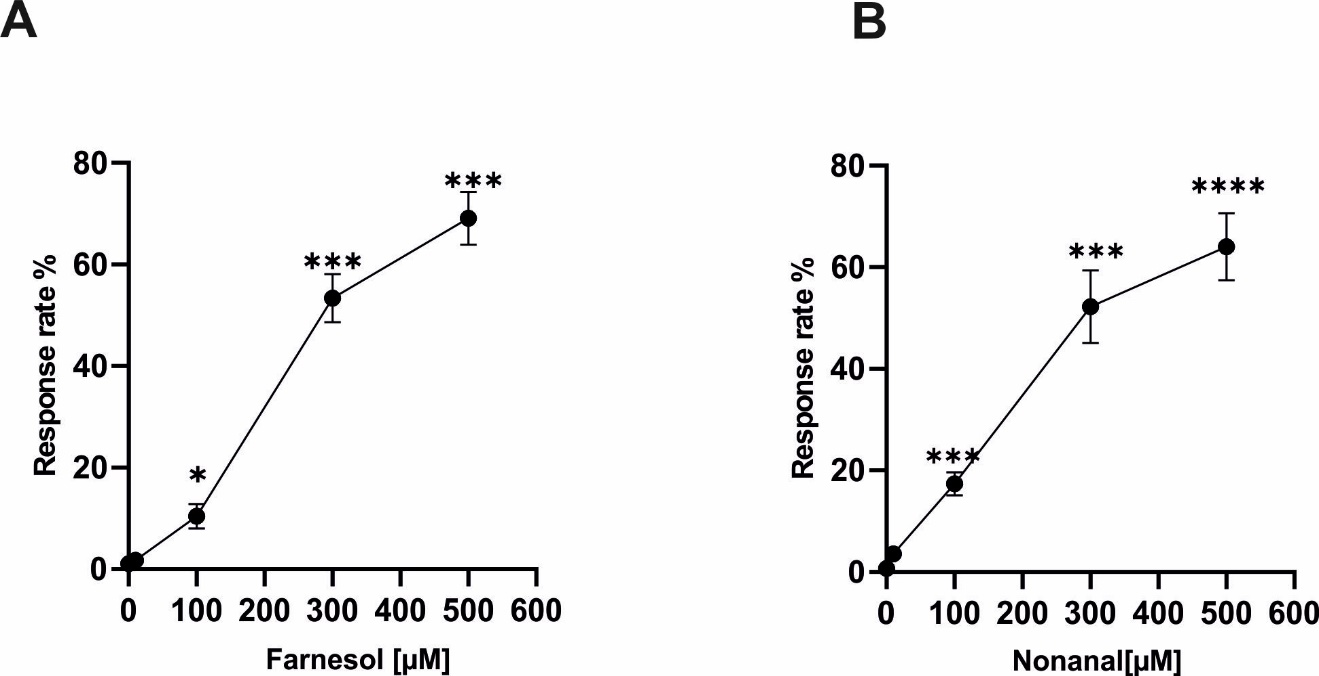


**Supplement Figure 1: Farnesol and Nonanal dose-dependently elevate the intracellular calcium levels.** The response rate of A549 to varying concentrations of (A) Farnesol and (B) Nonanal up to 500 µM, as well as to a solvent control of 0.1% DMSO, was quantified. Data were compared to the solvent using utilized One-way ANOVA. test with post hoc “Two-stage-up method of Benjamini, Krieger, and Yekutieli,”. *p ≤ 0.05, *** p ≤ 0.001and ****p ≤ 0.0001. TN = 9.


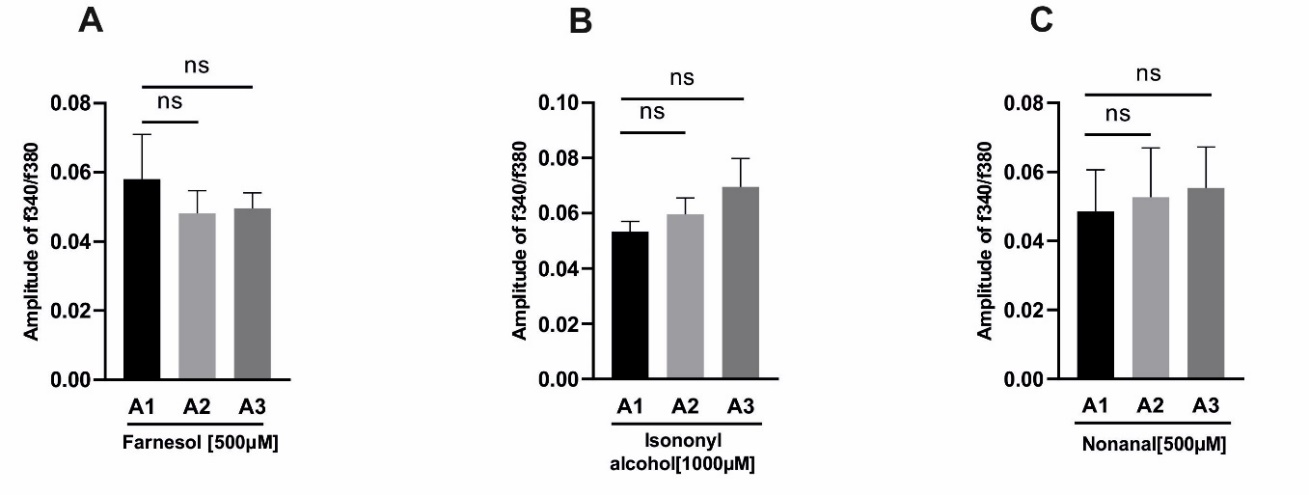


**Supplement Figure 2: Repetitive application of Farnesol, Isononyl alcohol and Nonanal do not affect short-term (de-) sensitization of ORs**. (A) Farnesol, (B) Isononyl alcohol and (C) Nonanal were applied 3 times for 1-3 minutes each. Concentrations are indicated on the graphs. Calcium imaging was used to assess the amplitude of calcium response, determined as the variance between f340/f380 wavelength readings. Data are shown as mean ± SEM, TN=9. Statistical significance vs. DMSO (0.1%) utilized One-way ANOVA. test with post hoc “Two-stage-up method of Benjamini, Krieger, and Yekutieli,” ns = non-significant.


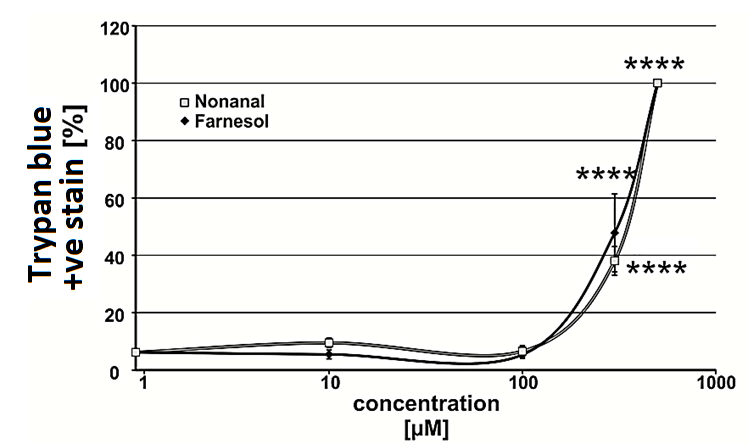


**Supplement Figure *3*: Famesol and Nonanal dose-dependently induce cell death in A549 cells.** A549 were exposed to increasing concentrations of Farnesol and Nonanal (10-500 µM) for 24h and cell viability was assessed by trypan blue dye exclusion. Cell toxicity was expressed as the ratio of Trypan blue positive cells against the total cell population. TN = 6. Data are shown as mean ± SEM. Statistical significance vs. DMSO (0.1%) utilized One-way ANOVA. test with post hoc “Two-stage-up method of Benjamini, Krieger, and Yekutieli,”. ****p ≤ 0.0001.


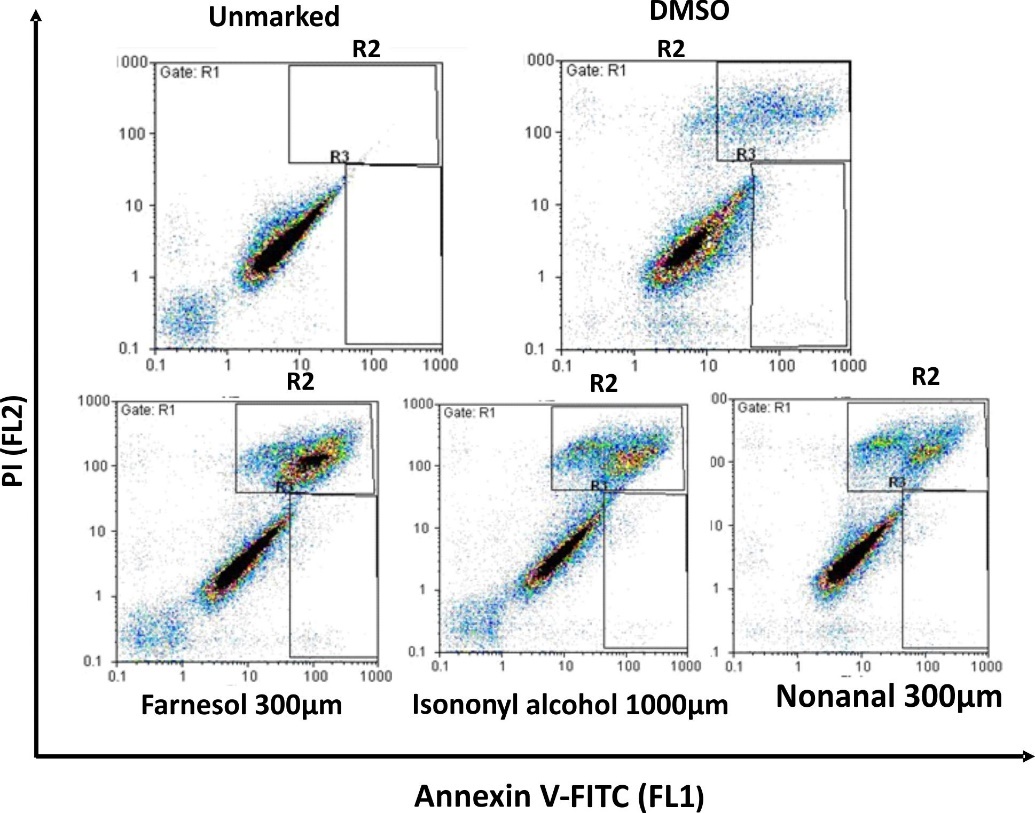


**Supplement Figure *4:* Stimulation of OR51B5 but not of OR1G1 reduces the viability of primary bronchial epithelial cells**. ALI-PBECs were stimulated with Farnesol, Isononyl alcohol or Nonanal vs solvent DMSO (0.1%). Proportions of viable, apoptotic, and necrotic cells were determined by flow cytometry using AV/ PI staining. AV+/ PI+ positive staining indicates necrotic cells referred to R2 (upper right quadrant) in the plot, TN = 5.

| Organ or  tissue | **Cell type** | **Olfactory receptors** | **Ligand**  **(endogenous/**  **exogenous** | **Dose in µM** | **Function** | **uses of Ligand** | **Refs** |
| --- | --- | --- | --- | --- | --- | --- | --- |
| Lung | human airway (SMCs) | **OR2AG1** | Amyl butyrate | 300 | Inhibition of the histamine-induced contraction of HASMCs | Synthetic odor used in perfumes | Kalbe et al., 2016) |
| Lung | human airway (SMCs) | **OR1D2** | Bourgeonal | 300 | increase:  contractility in (HASMCs)  secretion of IL-8 and GM-CSF | food industry  perfumes | Kalbe et al., 2016) |
| Skin | keratinocytes | **OR10G7** | Eugenol | 100 | Up regulated in atopic dermatitis patients  Increase Expression of IL-1ß mRNA | perfume  flavoring  local antiseptic | (Tham et al., 2019) |
| Eye | Retinal pigment epithelial (RPE) cells. | **OR51E2** | ß- ionone | 300 | increase the migration and proliferation of RPE cells | fragrance in cosmetic perfume and soaps | (Jovancevic et al., 2017) |
| GIT | Intestinal enteroendocrine L cell line (NCI-H716) | **OR51E1** | Nonanoic acid | 100 | Enhances GLP-1 and PYY secretion | Endogeno-usly secreted fatty acid | (Han et al., 2018) |
| Skin | Melanocytes  Keratinocyte | **OR2A4/7** | Cyclohexyl salicylate | 500 | Enhance melanin biosynthesis.  induces Apoptosis  of melanocytes  influences cell proliferation  secretion of IL-1 | Fragrance in cosmetics  household cleaners | (Wojcik et al., 2018)  (Tsai et al., 2017) |
| Kidney | Whole kidney tissue | **OR2T10** | Terpinyl acetate | 50-100 |  | Perfumes  foods  medicine | (Chowdhury & Kumar, 2020; Shepard, 2021) |
| Cardio-  vascular  system | Aorta-  coronary artery  umbilical vein endothelial cells (HUVEC) | **OR10J5** | Lyral | 300 | Enhanced angiogenesis  Stimulates cellular migration of HUVEC | Fragrance in cosmetics  Perfumes  and soap | (S. H. Kim et al., 2015)  (Hausen & Bottenbruch, n.d.) |
| Lung | GPCR-based biosensors in yeast cells | **OR2A7**  **OR10S1** | Lilial | 200-400 |  |  | (Yasi et al., 2019)  Kerslake et al., 2020) |
|  |  | **OR2T4** | Undecanal | 200 |  | Perfumes  deodorant | (Yasi et al., 2019)  (Park, 2022)  (Kalbe et al., 2016) |
| Blood  Skin | chronic myelogenous leukemia (CML) cell line K562  White blood cell in acute myelogenous leukemia (AML)  keratinocytes | **OR51B5** | Isononyl alcohol  Farnesol | 300- | Inhibit of proliferation of K562 cells.  Enhance keratinocyte migration.  IL-6 secretion | Fragrance products:  perfumed  deodorant | (Manteniotis et al., 2016)  (Tsai et al., 2017) ,Yasi et al., 2019) |
| Hair  Lung | Dermal papilla cells (DPCs)  A549  human alveolar cells | **OR1G1** | Nonanal  Nonanal | 100 | Promote DPC proliferation and migration.  Early and late apoptosis | Perfume  Food  additives | (Park et al., 2020)    (Choi et al. 2013) |

**Supplement Table 1. Screening of ORs in A549 cell line by different odorants on using** **real-time fluorometric calcium imaging.** A549 cells were loaded for 30 minutes with Fura 2-AM followed by measurement of intracellular calcium increase (f340nm/380nm ratio) in presence of odorant by three times application each for 3 min. The specific doses of odorants used in the experiments were determined based on previous literature and are outlined in the table. ATP (100 µM) was used as a positive control to ensure cell viability. Technical replicates (TN=6-9).
